# The DNA methyltransferase DNMT3A contributes to autophagy long-term memory

**DOI:** 10.1080/15548627.2020.1816664

**Published:** 2020-09-14

**Authors:** Patricia González-Rodríguez, Mathilde Cheray, Jens Füllgrabe, Maria Salli, Pinelopi Engskog-Vlachos, Lily Keane, Virginia Cunha, Agata Lupa, Wenbo Li, Qi Ma, Kristian Dreij, Michael G. Rosenfeld, Bertrand Joseph

**Affiliations:** a Institute of Environmental Medicine, Toxicology Unit, Karolinska Institutet, Stockholm, Sweden; b Department of Oncology-Pathology, Cancer Centrum Karolinska, Karolinska Institutet, Stockholm, Sweden; c Department of Medical Genetics, Cambridge Institute for Medical Research, University of Cambridge, Cambridge, UK; d Institute of Environmental Medicine, Biochemical Toxicology Unit, Karolinska Institutet, Stockholm, Sweden; e Howard Hughes Medical Institute, Department of Medicine, School of Medicine, University of California, San Diego, California, USA

**Keywords:** Autophagy, DNA methylation, epigenetics, MAP1LC3, transcription

## Abstract

Macroautophagy/autophagy is a conserved catabolic pathway that targets cytoplasmic components for their degradation and recycling in an autophagosome-dependent lysosomal manner. Under physiological conditions, this process maintains cellular homeostasis. However, autophagy can be stimulated upon different forms of cellular stress, ranging from nutrient starvation to exposure to drugs. Thus, this pathway can be seen as a central component of the integrated and adaptive stress response. Here, we report that even brief induction of autophagy is coupled *in vitro* to a persistent downregulation of the expression of MAP1LC3 isoforms, which are key components of the autophagy core machinery. In fact, DNA-methylation mediated by *de novo* DNA methyltransferase DNMT3A of *MAP1LC3* loci upon autophagy stimulation leads to the observed long-term decrease of *MAP1LC3* isoforms at transcriptional level. Finally, we report that the downregulation of MAP1LC3 expression can be observed *in vivo* in zebrafish larvae and mice exposed to a transient autophagy stimulus. This epigenetic memory of autophagy provides some understanding of the long-term effect of autophagy induction and offers a possible mechanism for its decline upon aging, pathological conditions, or in response to treatment interventions.

**Abbreviations:** ACTB: actin beta; ATG: autophagy-related; 5-Aza: 5-aza-2’-deoxycytidine; BafA1: bafilomycin A_1_; CBZ: carbamazepine; CDKN2A: cyclin dependent kinase inhibitor 2A; ChIP: chromatin immunoprecipitation; Clon.: clonidine; CpG: cytosine-guanine dinucleotide: DMSO: dimethyl sulfoxide; DNA: deoxyribonucleic acid; DNMT: DNA methyltransferase; DNMT1: DNA methyltransferase 1; DNMT3A: DNA methyltransferase alpha; DNMT3B: DNA methyltransferase beta; dpf: days post-fertilization; EBSS: Earle’s balanced salt solution; EM: Zebrafish embryo medium; GABARAP: GABA type A receptor associated protein; GABARAPL1: GABA type A receptor associated protein like 1; GABARAPL2: GABA type A receptor associated protein like 2; GAPDH: glyceraldehyde-3-phosphate dehydrogenase; GRO-Seq: Global Run-On sequencing; MAP1LC3/LC3: microtubule-associated protein 1 light chain 3; MAP1LC3A: microtubule-associated protein 1 light chain 3 alpha; MAP1LC3B: microtubule-associated protein 1 light chain 3 beta; MAP1LC3B2: microtubule-associated protein 1 light chain 3 beta 2; MEM: minimum essential medium; MEF: mouse embryonic fibroblasts; mRNA: messenger RNA; MTOR: mechanistic target of rapamycin kinase; PBS: phosphate-buffered saline; PIK3C3: phosphatidylinositol 3-kinase catalytic subunit type 3; RB1CC1/FIP200: RB1 inducible coiled-coil 1; RT-qPCR: quantitative reverse transcription polymerase chain reaction; SQSTM1/p62: sequestosome 1; Starv.: starvation; Treh.: trehalose; ULK1: unc-51 like autophagy activating kinase 1.

## Introduction

Macroautophagy, referred to throughout as autophagy, is a conserved catabolic pathway in which portions of the cytoplasm such as damaged organelles, misfolded proteins and intracellular pathogenic bacteria are delivered to lysosomes to be degraded and recycled. This biological process is a tightly regulated pathway involving a series of dynamic membrane-rearrangements orchestrated by a core set of autophagy-related (ATG) proteins [1]. Autophagy involves the assembly of the phagophore, followed by the formation of the autophagosome that contains the cargo to be degraded. Subsequently, autophagosomes fuse with the lysosomes (generating autolysosomes) breaking down the cargo by lysosomal enzymes and eventually the macromolecules generated are then reused in a wide range of cellular processes [2,3,4].

In order to ensure cell homeostasis, a constitutive basal level of autophagy is required under physiological conditions [5]. However, upon extra- or intracellular stress such as starvation, hypoxia, DNA damage, endoplasmic reticulum stress, pathogen infection or exposure to pharmaceutical drugs, auto-phagy is upregulated [6]. Following stress conditions, cells adapt and return to their basal level of autophagy by fine-tuned regulation of this process, which extends from epigenetic and transcriptional level to post-translational protein modifications [7].

Recent studies revealed a role for several histone modifications and histone-modifying enzymes in the regulation of autophagy, thereby uncovering the existence of an epigenetic network controlling this process [8,9,10]. The identification of histone modifications associated with the autophagic process offers an attractive conceptual framework to understand the short-term transcriptional response to stimuli eliciting autophagy, as well as constituting a potential component for long-term responses to autophagy [11,12,13]. However, histone post-translational modifications are considered to primarily promote reversible repression of specific genes, while DNA methylation would contribute to long-lasting effects [14,15]. Therefore, it is interesting to speculate that DNA methylation, possibly directed by prior histone post-translational modifications, could promote heritable changes in gene expression in cells exposed to cellular stress such as an autophagy induction.

DNA methylation is regulated by DNA methyltransferase (DNMTs) enzymes that catalyze the transfer of a methyl group to the fifth carbon of a cytosine ring in cytosine-guanine dinucleotide (CpG) dinucleotides generating 5-methylcytosine (5mC) [16,17]. DNA methylation is a dynamic and reversible process that include three possible phases: establishment (*de novo* DNA methylation), maintenance and demethylation. In mammals, the establishment of novel cytosine methylation profiles in cells is carried out by two *de novo* DNA methylation enzymes, DNMT3A (DNA methyltransferase 3 alpha) and DNMT3B (DNA methyltransferase 3 beta). However, the maintenance of these CpG methylation patterns in the daughter cell upon DNA replication depends on the activity of another DNMT, *i.e*., the methylation maintenance enzyme DNMT1 (DNA methyltransferase 1) that acts in concert with its cofactor UHRF1 (ubiquitin-like with PHD and RING finger domains 1). Active DNA demethylation is performed by the TET methylcytosine dioxygenases, which progressively oxidize 5mC [18,19].

A hallmark of autophagy is the formation of autophagosomes that engulfs and degrades cytosolic components via fusion with the lysosome. The Atg8 protein family (named after yeast Atg8) are essential for autophagosome biogenesis/maturation, and also exert functions as adaptor receptor proteins for selective autophagy. In mammals, there are two subgroups of Atg8-like proteins: MAP1LC3 (microtubule-associated protein light chain 3) proteins, including MAP1LC3A, MAP1LC3B, and MAP1LC3C, and GABARAPs (GABA type A receptor-associated proteins), including GABARAP, GABARAPL1, and GABARAPL2. Despite some aspect of functional redundancy, the members of the two protein families have been convincingly shown to exert different roles in the spatiotemporal regulation of autophagy [20,21,22,23]. As an illustration, it is reported that deficiency in the entire MAP1LC3 family results in defects in the autophagic flux, which is not compensated by the presence of the GABARAP family [24].

In this study, we reveal that cell exposure to even brief autophagic stimuli is associated with an upregulation of DNMT3A that leads to a heritable and stable increased DNA methylation pattern on selected autophagy-related genes. In fact, this epigenetic memory of autophagy based on DNA methylation promotes a prolonged decrease in the basal autophagy level through a persistent downregulation of MAP1LC3, central proteins in the autophagy pathway, in the previously autophagy-exposed cells.

## Results

### Autophagy induction results in persistent downregulation of MAP1LC3 expression levels

**Figure 1.**
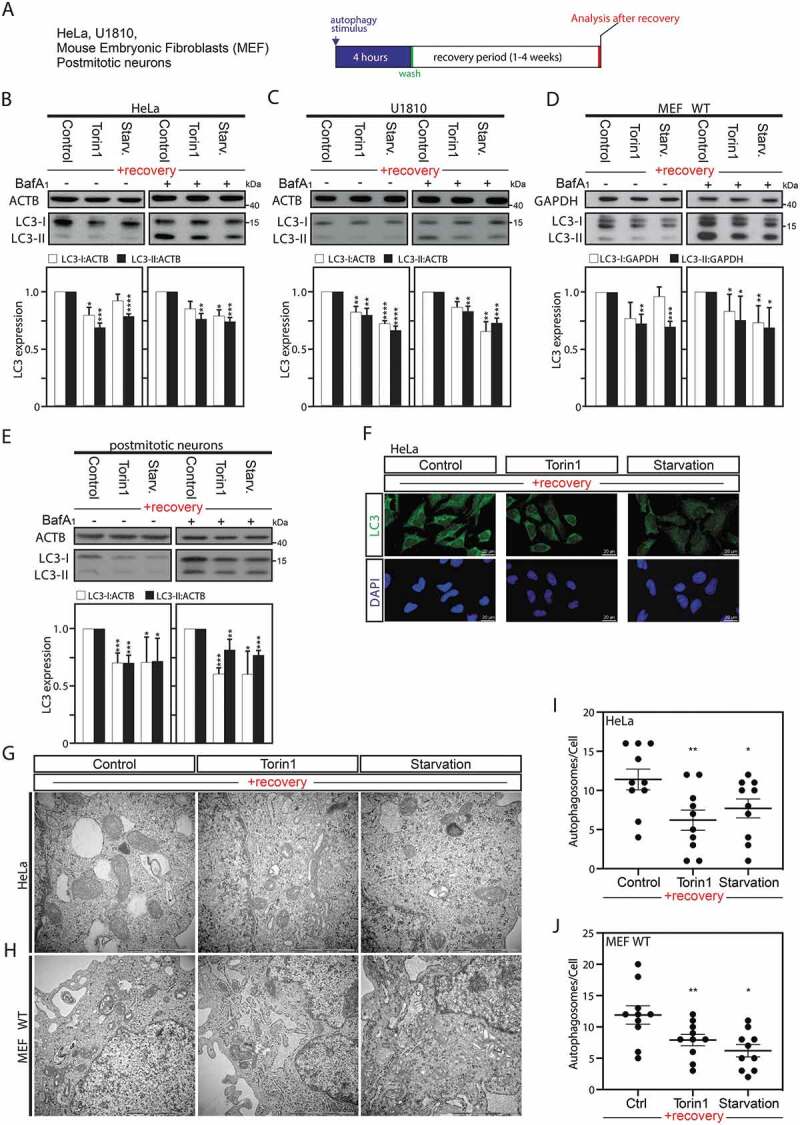
Autophagy induction is associated with a sustained downregulation of MAP1LC3 protein expression after a recovery period. (A) Illustrative pipeline of the “previously-autophagy exposed cells” generation and analysis after a recovery period. (B–E) Immunoblot analysis and quantification of LC3-I and LC3-II expression versus ACTB or GAPDH in HeLa cells (B), U1810 cells (C), wild-type (WT) MEF cells (D), or post-mitotic neurons (E), starved (Starv.), pretreated with torin1, or DMSO (used as control) for 4 h and thereafter left to recover and analyze after 2 weeks (B and C), one week (D), or 8 d (E) under normal cell culture conditions. Treatment with the late inhibitor of autophagy bafilomycin A_1_ (BafA1) before sample collection validates that the observed overall decrease in LC3 expression was not the result of an increase in autophagic flux (B to E). (F) Immunofluorescence confocal microscopy imaging of endogenous LC3 in HeLa cells after 2-week recovery period. (G to J) Ultrastructural analysis by electron microscopy of HeLa cells (G and I) or MEF cells (H and J), starved, pretreated with torin1, or DMSO (used as control) for 4 h and thereafter left to recover and analyze after 2 weeks (HeLa cells), or one week (MEF cells). Panels I and J are quantification of number of autophagosomes per cell. 10 cells were counted per conditions. All values are a mean of at least 3 independent experiments ± SEM and considered significant for *p < 0.05, **p < 0.01, ***p < 0.001, ****p < 0.0001. n.s., not significant for the indicated comparison. (B-E, n = 3–5; I-J, n = 10)

To investigate long-lasting effects of autophagy, we generated a panel of “previously autophagy-exposed cell lines,” *i.e*., HeLa or U1810 cancer cells and mouse embryonic fibroblasts (MEFs) transiently exposed to an autophagy stimulus (*i.e*., amino acid starvation or treatment with torin1) for 4 h (a time point where significant autophagy induction could be confirmed), and then left to recover under normal culture condition from 1 to 4 weeks. After this recovery period, these cells previously exposed to autophagy as well as their parental counterparts used as controls (non-autophagy-exposed cells or treated with DMSO [dimethyl sulfoxide]) were used to investigate differences in baseline autophagy after recovery (Figures 1A and S1).

Before investigating this biological process in cells that recovered from exposure to an autophagic stimulus, we first validated that the cells responded to this initial stimulus (Fig. S2A). The occurrence of autophagy was measured by an increased lipidation of MAP1LC3, referred as LC3-II, in these cells (Fig. S2B-F). Furthermore, we ascertained that autophagy induction was not linked to an increase in cell death in HeLa cells, and thus that the observed effects in those cells previously exposed to an autophagy stimulus, would not be the result of a selection of a cell sub-population after the first stimuli (Fig. S2G).

After the recovery period, the previously autophagy-exposed cell lines, as compared to their parental counterparts, appeared to differ in their expression levels of this autophagic marker. Indeed, after one-week (MEF) or two weeks (HeLa and U1810 cells) recovery time, a decrease in the basal LC3 levels was observed in cells previously exposed to an autophagic stimuli (Figure 1B–D). Likewise, after autophagy induction for 4 h in post-mitotic non-dividing mouse cortical neurons, a reduction in LC3 expression levels was observed at 8 d recovery time, indicating that the observed effect is not the result of the selection of a subpopulation from a proliferative cell culture over the recovery period (Figures 1E and S2E). MTOR (mechanistic target of rapamycin kinase) is involved in a wide variety of signaling pathways and therefore, treating with an MTOR inhibitor (such as torin1) or starving the cells could cause the observed changes by mechanisms unrelated to autophagy [25]. To exclude this possibility, HeLa cells were treated with carbamazepine, trehalose or clonidine, MTOR-independent inducers of autophagy, which also led to a similar reduction in LC3 protein expression levels (Fig. S2F, S3A and S3B). Bafilomycin A_1_ (BafA_1_) is a late inhibitor of autophagy that prevents the fusion between autophagosomes and lysosomes and thus blocks LC3-II/autophagosome degradation. BafA_1_ co-treatment experiments were performed to confirm the total reduction in LC3 protein expression level *per se* in cells previously exposed to an autophagic stimulus MTOR-dependent and – independent compared to the non-autophagy-exposed cells (Figures 1B–E and S3A). Moreover, decreased endogenous LC3 expression was further confirmed by immunofluorescence analysis in HeLa cells previously exposed to MTOR-dependent and MTOR-independent inducers of autophagy (Figures 1F and S3B).

Finally, we performed ultrastructural analysis, using electron microscopy, after a recovery period of previously autophagy-exposed HeLa (Figure 1G,I) and MEF cells (Figure 1H,J). Electron microscopy, which is considered as one of the most accurate methods for the detection of autophagosome formation, revealed in both cell types a significant decrease in the number of autophagosome in cells previously exposed to an autophagy stimulus as compared to unstimulated cells (Figure 1G–J). Collectively those data indicate that the observed reduction in LC3 protein levels in the previously autophagy-exposed cells is the result of the downregulation of its expression rather than an increase of its degradation, which is then associated with a reduced number of autophagosomes after recovery.

### Short autophagy induction has an impact on the expression of MAP1LC3 genes

Consequently, we wondered whether the persistent downregulation of the LC3 protein level in cells where autophagy was induced either by starvation or by chemical inducers was due to transcriptional regulation. For this purpose, by using the same model as previously described in detail in Figure 1A, we checked *MAP1LC3* mRNA expression levels after a recovery period in U1810, HeLa and MEF Cells.

**Figure 2.**
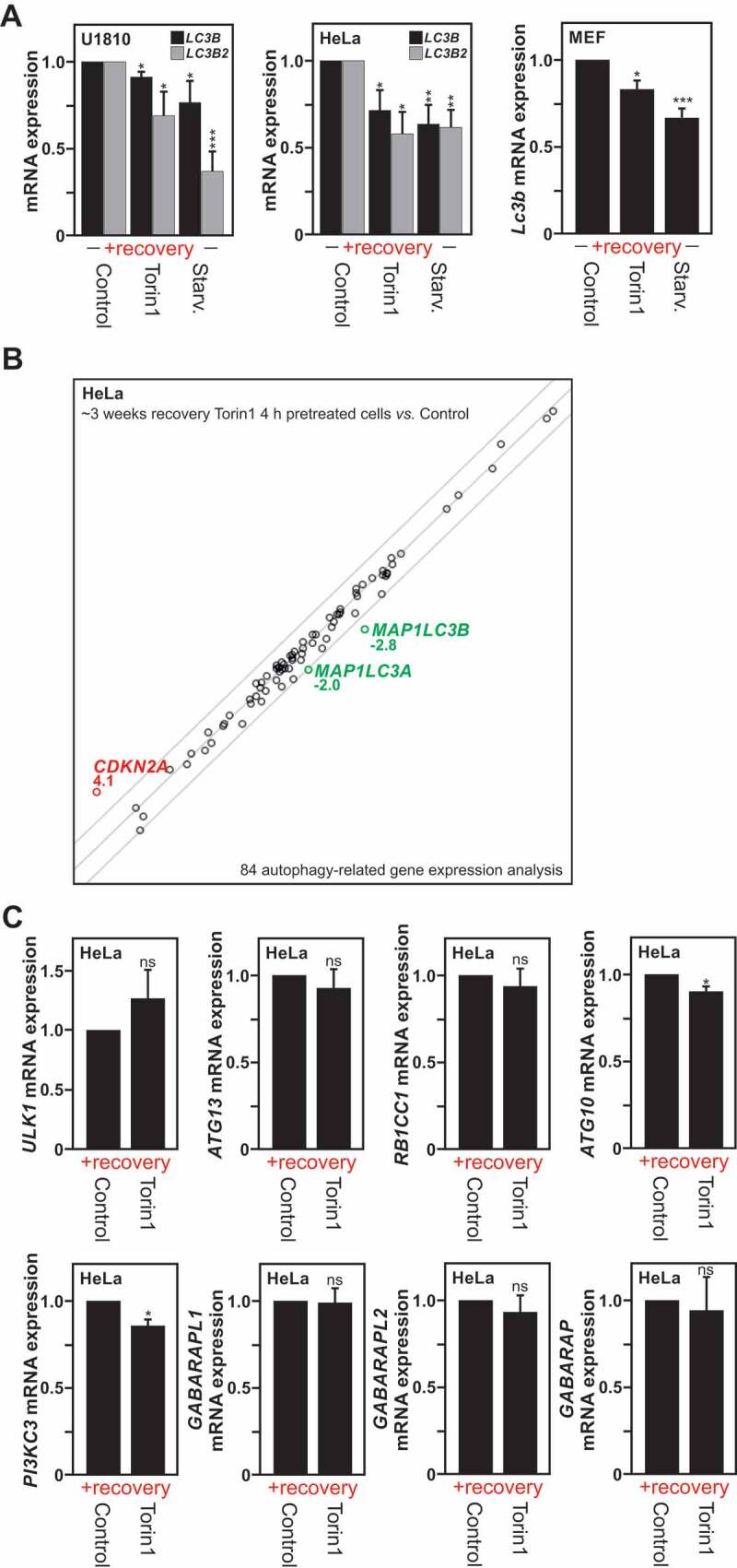
Transcriptional repression of the *MAP1LC3* genes is observed upon autophagy induction. (A) *MAP1LC3B* and *MAP1LC3B2* isoforms mRNA expression measured by RT-qPCR in previously autophagy-exposed U1810, HeLa and MEF cells processed as described in Fig. S1A and analyzed after a specific recovery period. (B) Analysis of mRNA expression of 84 autophagy-related genes (from a RT-qPCR based gene array) in HeLa cells following a 3 weeks recovery-period after an initial torin1 or DMSO (used as control) treatment for 4 h. Representation of n = 3 independent experiments. (C) Analysis by RT-qPCR of mRNA expression of *ATG10, ATG13, PIK3C3, RB1CC1, ULK1, GAPARAPL1, GABARAPL2* and *GABARAP* genes in HeLa cells processed as described in panel B. All values are means of at least 3 independent experiments ± SEM and considered significant for *p < 0.05, **p < 0.01, ***p < 0.001. n.s., not significant for the indicated comparison. (A, n = 4–6; B and C, n = 3)

*MAP1LC3B* mRNA expression levels per se were found to be significantly reduced in cells previously exposed to either starvation or treatment with torin1, trehalose or clonidine, as compared to their untreated counterparts, and *MAP1LC3B2* mRNA expression was found affected in cells previously exposed to starvation, torin1 or trehalose (Figures 2A and S3D). In contrast, exposure to Carbamazepine, a voltage-dependent sodium channels blocker, was not found to be associated with the downregulation of these *MAP1LC3* genes after the same recovery period (2 weeks). This observation might not be surprising considering it could have an additional variety of mechanisms of action compared to different autophagy inducers used in the study, some of which that could include compensatory mechanism.

To obtain a deeper understanding of the long-term impact of an initial autophagy stimulus on autophagy-related gene expression, we took advantage of the Qiagen human autophagy RT2 profiler polymerase chain reaction (PCR) array that monitors the expression of 84 genes involved in the machinery and regulation of autophagy (**supplementary data file**). Hence, we investigated possible differences in the mRNA expression of these genes in HeLa cells left to recover under normal culture conditions for 3 weeks period after an initial transient stimulation for 4 h. The expression levels of most transcripts were found to be mostly unaltered after this recovery period. However, the mRNA expression levels of three genes, *CDKN2A* (cyclin dependent kinase inhibitor 2A) (<2-fold increase), *MAP1LC3A* and *MAP1LC3B* (>2-fold decrease) were found to be significantly altered (Figure 2B). Besides the three genes pointed out, *i.e. MAP1LC3A, MAP1LC3B* and *CDKN2A*, three additional genes were close to the threshold lines in the human autophagy RT2 profiler PCR array, namely *ATG10, GABARAPL2*, and *PIK3C3* (Phosphatidylinositol 3-kinase catalytic subunit type 3). Hence, their mRNA expression levels were further investigated by RT-qPCR. The expression of *ATG10* and *PIK3C3* genes were also found to be significantly reduced after the recovery period. However, neither *GABARAPL2, GABARAPL1* or *GABARAP* mRNA expression levels were not found to be significantly regulated by RT-qPCR analysis (Figures 2C and 4C). Likewise, the expression of *ULK1* (unc-51 like autophagy activating kinase 1), *ATG13*, and *RB1CC1/FIP200* (RB1 inducible coiled-coil 1) genes, whose encoded proteins products participate in the ULK1/Atg1 complex, which plays an essential role in the initiation of autophagy were not found to be affected (Figure 2C). Altogether, these results revealed that even transient autophagy induction is associated with a long-lasting and mainly selective transcriptional repression of *MAP1LC3*, which account for the above described decrease in LC3 protein expression levels. Of note, other genes such as *ATG10* or *PIK3C3* were also found to be downregulated, which might contribute to the observed decrease in baseline autophagy after recovery in those cells previously exposed to an autophagy stimulus

### DNMT3A recruitment and DNA methylation on MAP1LC3 promoters upon autophagy induction

**Figure 3.**
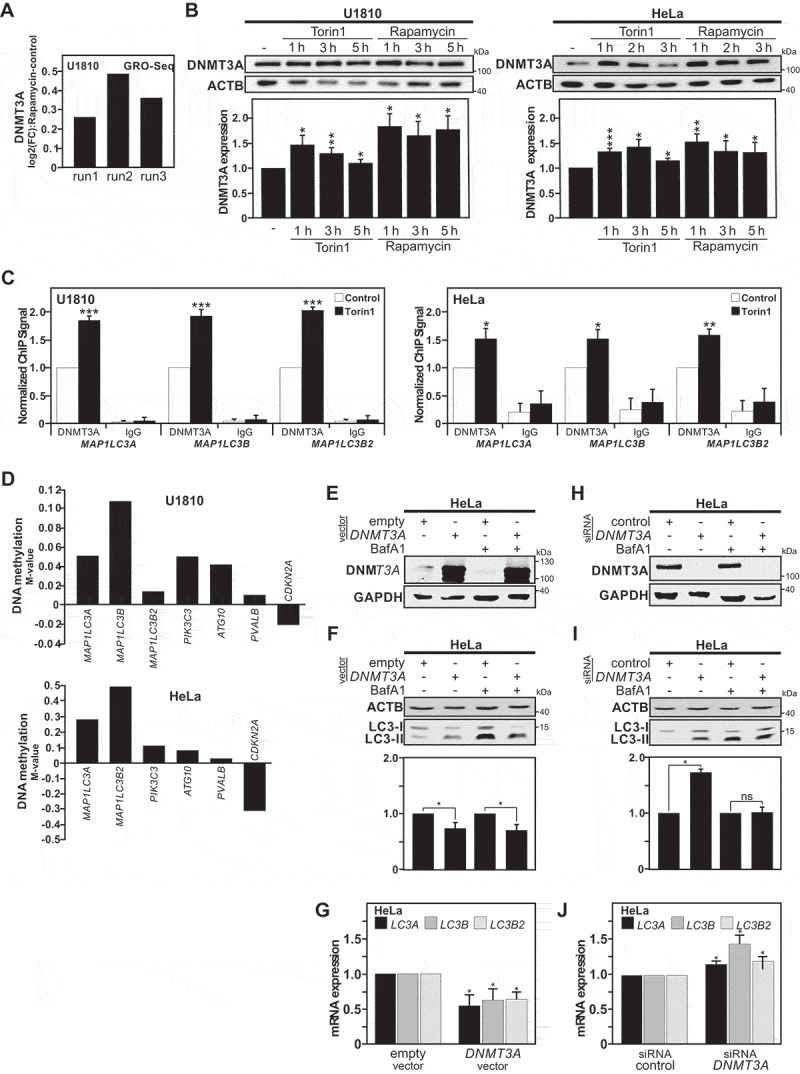
DNMT3A recruitment and DNA methylation occur at the *MAP1LC3* loci in response to autophagy stimulation. (A) Upregulation of DNMT3A gene transcription upon autophagy induction was found in previously published GRO-seq (Global Run-On Sequencing) analysis of rapamycin-treated U1810 cells for 2 or 8 h [8]. Data are presented as log2 (fold-change) (log2[FC]) of normalized unit of *DNMT3A* transcript expression for rapamycin-treated cells versus control cells, for each independent experimental replicate/run (n = 3). (B) Immunoblot analysis of DNMT3A protein expression in U1810 and HeLa cells upon rapamycin or torin1 treatments at indicated time points. The graphs show quantification for DNMT3A versus ACTB expression in Hela and U1810 cells when compared to the DMSO treated ones (used as control). Representation of n = 3 independent experiments; mean ± SEM. (C) Chromatin Immunoprecipitation (ChIP) analysis of DNMT3A recruitment on the *MALP1LC3* isoforms loci upon induction of autophagy with torin1 in HeLa Cells for 6 h and U1810 cells for 2 h. (D) Analysis of individual CpG site methylation level (using the M-value normalized with the control samples, *i.e*., log2 ratio of methylated:unmethylated probe intensity) at the *ATG10, CDKN2A, PIK3C3, PVALB* and *MAP1LC3* isoforms loci in cells previously exposed to an autophagy stimulus when compared with non-stimulated control cells (M-value = 0) after 3 weeks recovery period. (E and F) Immunoblot analysis of DNMT3A in HeLa cells upon transfection for 24 h with *DNMT3A* plasmid or an empty vector (used as control). (E) Immunoblot of LC3-II expression in HeLa cells upon DNMT3A overexpression with and without bafilomycin A_1_ (BafA1) treatment and (F) quantification of total LC3 expression (LC3-I and LC3-II) versus ACTB levels. (G) Analysis of mRNA expression of *MAP1LC3* isoforms when DNMT3A is overexpressed in HeLa Cells compared to the empty vector (used as control) after 24 h post-transfection. (H) Immunoblot of DNMT3A in HeLa cells transfected with a pool of siRNAs against *DNMT3A* compared with siRNAs control with and without BafA1 treatment and (I) Quantification of total LC3 expression versus ACTB levels. (J) RT-qPCR analysis of MAP1LC3 isoforms expression in HeLa cells upon *DNMT3A* silencing compared to control. All values are means of at least 3 independent experiments ± SEM and considered significant for *p < 0.05, **p < 0.01, ***p < 0.001. n.s., not significant for the indicated comparison

Thereafter, we extended our investigation to the exploration of the molecular mechanism that could account for the persistent transcriptional repression of *MAP1LC3*. Therefore, we examined DNA methylation that can stably affect the expression of genes in a long-term manner and is usually heritable through each mitotic cell division [26,27]. Firstly, we performed re-analysis of data obtained from Global Run-On sequencing (GRO-seq) of rapamycin-treated U1810 cells (ArrayExpress accession number E-MTAB-5490). This method provides a genome-wide quantitative snapshot of transcriptional activity in cells in response to an autophagy stimulus. Noteworthy, we found a consistent upregulation of *DNMT3A* gene transcription upon autophagy induction (Figure 3A). Upon autophagy induction, DNMT3A upregulation at protein level, as well as its accumulation in the nucleus was observed (Figures 3B, S4A and S4B). Expression of DNMT3B and DNMT1 were also investigated upon autophagy induction in both cell lines but not found to be significantly affected (Fig. S4C). To gain additional molecular insight into the role of DNMT3A in the regulation of *MAP1LC3* genes, the targeted recruitment of DNMT3A at the *MAP1LC3* loci was investigated by chromatin immunoprecipitation (ChIP). Shortly after the initial exposure to torin1, accumulation of DNMT3A protein was observed at the potential CpG sites in the *MAP1LC3A, MAP1LC3B* and *MAP1LC3B2* genes (Figure 3C). Notably, accumulation of DNMT3A protein was also observed at the potential CpG sites in the *ATG10* and *PIK3C3* genes (Fig. S5A). In contrast, increased occupancy for DNMT3B on those genomic regions of the *MAP1LC3* genes was not observed, as assayed by DNMT3B ChIP (Fig. S5B). This observation proposes that upon short autophagy induction, DNMT3A is recruited to the promoter of the *MAP1LC3* isoforms and might lead to methylation of these DNA regions, accounting for the previously found persistent downregulation of LC3 at mRNA and protein level after recovery.

In order to address whether the observed regulation of DNMT3A upon autophagy induction could result in persistent changes in DNA methylation patterns, we used the Illumina® Infinium HumanMethylation450 BeadChip array. Thus, we compared the genome-wide DNA methylation profiles of cells transiently exposed to torin1 or DMSO for 4 h and then left to recover for a 4 weeks period (Figure 3D). After this recovery period, analysis of global DNA methylation levels showed no difference between torin1 or DMSO pre-treated cells (Fig. S5C). However, in agreement with the idea that even short treatment with autophagy inducers could promote persistent epigenetic changes in specific genes, DNA methylation patterns were found to cluster according to the pre-treatment (DMSO versus torin1) instead of harvesting time points (biological repeats) (Fig. S5D). Furthermore, locus-specific analysis revealed increased DNA methylation at CpG sites across the *MAP1LC3* loci after recovery (Figure 3D). Reduced DNA methylation was observed for the *CDKN2A* locus whereas increased DNA methylation was observed for the *ATG10* and *PIK3C3* loci in agreement with the potential regulation of their gene expressions (Figures 2C and 3D). To further illustrate that the timing aspect of the described phenomena, *i.e*., autophagy induction and subsequent transcriptional downregulation of the expression of *MAP1LC3* genes by DNMT3A, we performed timeline experiments using torin1-treated cells and LC3 conversion by immunoblot, whereas *MAP1LC3B* gene expression was monitored by RT-qPCR. These experiments confirmed that the repression of the *MAP1LC3B* gene expression occurred secondary to induction of autophagy (Fig. S6A and S6B). These results support the concept that DNMT3A-mediated DNA methylation of the *MAP1LC3* loci provides an epigenetic and heritable mechanism for the long-term regulation of MAP1LC3 expression upon autophagy induction.

In order to gain further understanding of the DNMT3A-dependent regulation of *MAP1LC3* gene expression, we modulated DNMT3A expression levels and investigated the impact on *MAP1LC3* gene expression. In agreement with the observed effect of autophagy-induced DNMT3A upregulation on *MAP1LC3B* gene expression, a significant decrease in global MAP1LC3B protein expression levels was observed in DNMT3A-overexpressing HeLa cells in which the autophagy and thus LC3-II degradation have been blocked by BafA_1_ treatment (Figure 3E,F). In fact, the overexpression of DNMT3A appeared to negatively impact on the mRNA expression of *MAP1LC3A, MAP1LC3B* and *MAP1LC3B2* isoforms (Figure 3G). In contrast, the repression of DNMT3A expression using a pool of siRNAs targeting *DNMT3A* positively affected the expression of LC3 at protein level as well as the mRNA expression of *MAP1LC3* isoforms in HeLa cells (Figures 3H,J, S6D and S6E). Furthermore, methylation-specific (MS)-PCR analysis showed that torin1 treatment-induced DNA methylation at the CpG sites of *MAP1LC3* was reduced in HeLa cells transfected with the *DNMT3A* siRNAs pool at 24 h, as compared to HeLa cells transfected with a control pool of siRNAs (Fig. S6C). RT-qPCR analysis revealed that knockdown of *DNMT3A* expression prior to autophagy induction, lowered torin1-induced decrease in *MAP1LC3* genes at 24 h, arguing for the role of DNMT3A in the downregulation of the *MAP1LC3* isoforms gene expression (Fig. S6D). Of note, LC3 immunoblot demonstrated that knockdown of *DNMT3A* expression in HeLa cells increased their LC3 protein expression level at baseline and upon stimulation with an autophagy inducer such as torin1 at different time points (Fig. S6E).

These observations pushed us to investigate whether a DNA demethylation-chemicals agent such as 5-aza-2ʹ-deoxycytidine (5-Aza) alone could promote the expression of *MAP1LC3* genes. For this purpose, HeLa cells were treated with two doses, *i.e*. 1 µM and 10 µM, of 5-Aza. In a dose-dependent manner, 5-Aza treatment was found to increase MAP1LC3 expression at both mRNA and protein levels, suggesting that the *MAP1LC3* loci exhibit some level of DNA methylation even under baseline conditions. (Fig. S6F-H). Collectively, these data support a transcriptional regulation of *MAP1LC3* isoforms by DNMT3A-mediated DNA methylation.

### MAP1LC3 downregulation has functional consequences for autophagy

**Figure 4.**
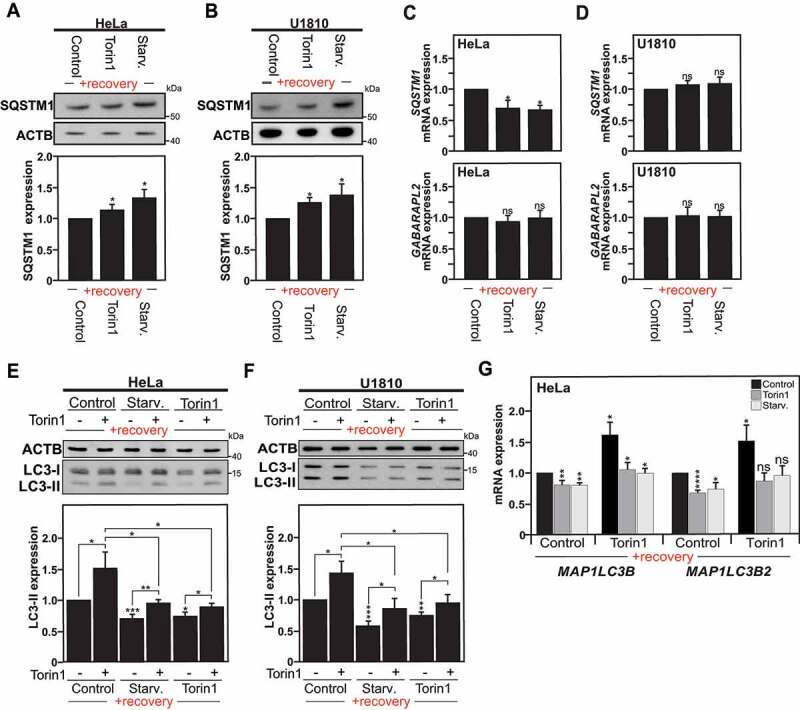
Autophagy is altered in cells previously exposed to an autophagy stimulus. (A and B) Immunoblot analysis of SQSTM1 expression in Hela cells (A), or U1810 cells (B), starved (Starv.), treated with torin1 or DMSO (used as control) for 4 h, and left to recover under normal culture conditions for a two to three weeks period. The graphs show the quantification of SQSTM1 versus ACTB expression in Hela cells (A), and U1810 cells (B). (C and D) *SQSTM1* and *GABARAPL2* mRNA expression levels measured by RT-qPCR in HeLa cells (C) and U1810 cells (D) previously exposed to an autophagy stimulus or DMSO (used as control) and analyzed at 2 weeks after the first autophagy stimulus. (E and F) Immunoblot and quantification analysis of LC3-II expression in Hela cells (E), or U1810 cells (F), previously exposed to an autophagy stimulus as described in Fig. S1A upon re-stimulation of autophagy with torin1 treatment for 1 h (+) as compared to DMSO treatment used as control (-). G *MAP1LC3B* and *MAP1LC3B2* isoforms mRNA expression measured by RT-qPCR in HeLa cells previously exposed to an autophagy stimulus upon re-stimulation of autophagy with torin1. All values are means of at least 3 independent experiments ± SEM and considered significant for *p < 0.05, **p < 0.01, ***p < 0.001. n.s., not significant for the indicated comparison. (n = 3–6)

In order to investigate the effect of long-term MAP1LC3 downregulation observed in the “previously autophagy-exposed cells” on the autophagy process *per se*, we looked at the expression levels of SQSTM1/p62 (sequestosome 1). SQSTM1 protein serves as a receptor that delivers ubiquitinated proteins to the proteasome for degradation and thereby links the ubiquitin-proteasome system and autophagy pathway [28,29,30]. Consequently, the fact that SQSTM1 is degraded by autophagy is used as common marker to study autophagy flux as autophagy inhibition usually coincides with increased levels of SQSTM1 due to reduced degradation [31]. In fact, a small but significant increase in SQSTM1 expression at protein level was observed in cells that were previously stimulated with either starvation or torin1 as compared to the control ones in HeLa (Figure 4A) and U1810 cells (Figure 4B). Noteworthy, it appears that the observed increase in SQSTM1 protein level was not the result of increased *SQSTM1* mRNA expression. Moreover, no compensatory increase in the expression of Atg8 family members, such as *GABARAPL2*, was observed (Figures 2C and 4C,D). These data confirm the persistent downregulation of baseline autophagy observed in previously autophagy-exposed cells.

Considering that reduced MAP1LC3 expression could act as a rate-limiting factor for the autophagy pathway, we decided to study whether the sustained downregulation of MAP1LC3 could affect the response of the cells to a new autophagy stimulus. Hence, after a recovery period, parental cells and cells previously exposed to either MTOR-dependent or MTOR-independent inducers were re-stimulated for 1 h with torin1 and MAP1LC3 protein expression was analyzed. Noteworthy, the cells that were re-stimulated with a second autophagic stimulus were able to undergo autophagy successfully but showed reduced LC3 protein level as compared to cells exposed to the same autophagic stimulus for the first time (Figures 4E,F and S3C).

Using the same conditions, we investigated whether the transcriptional responses for the *MAP1LC3B* and *MAP1LC3B2* genes, which were found to be the gene targets for the DNMT3A-mediated repressive effect upon autophagy induction, were found to be affected after re-stimulation with torin1 treatment for 1 h. Upon autophagy induction, the level of *MAP1LC3/ATG8* mRNA are reported to significantly increase [32,33,34,35]. Interestingly, in response to a novel autophagy stimulus, the gene expression induction for these two *MAP1LC3* isoforms was found to be robustly reduced in the cells previously exposed to an autophagic stimulus, as compared to the unstimulated cells (Figures 4G and S3D). Of note, our data supports the fact that DNMT3A-mediated persistent MAP1LC3 downregulation by DNA methylation does not completely suppress the expression of the *MAP1LC3* isoforms but rather decreases its expression. Interestingly, we found that cells previously exposed to an autophagic stimulus are able to undergo autophagy successfully compared with the non-stimulated ones, but they start with a lower baseline level.

### ATG7 deficiency prevents the acquisition of autophagy-induced long-term effects

**Figure 5.**
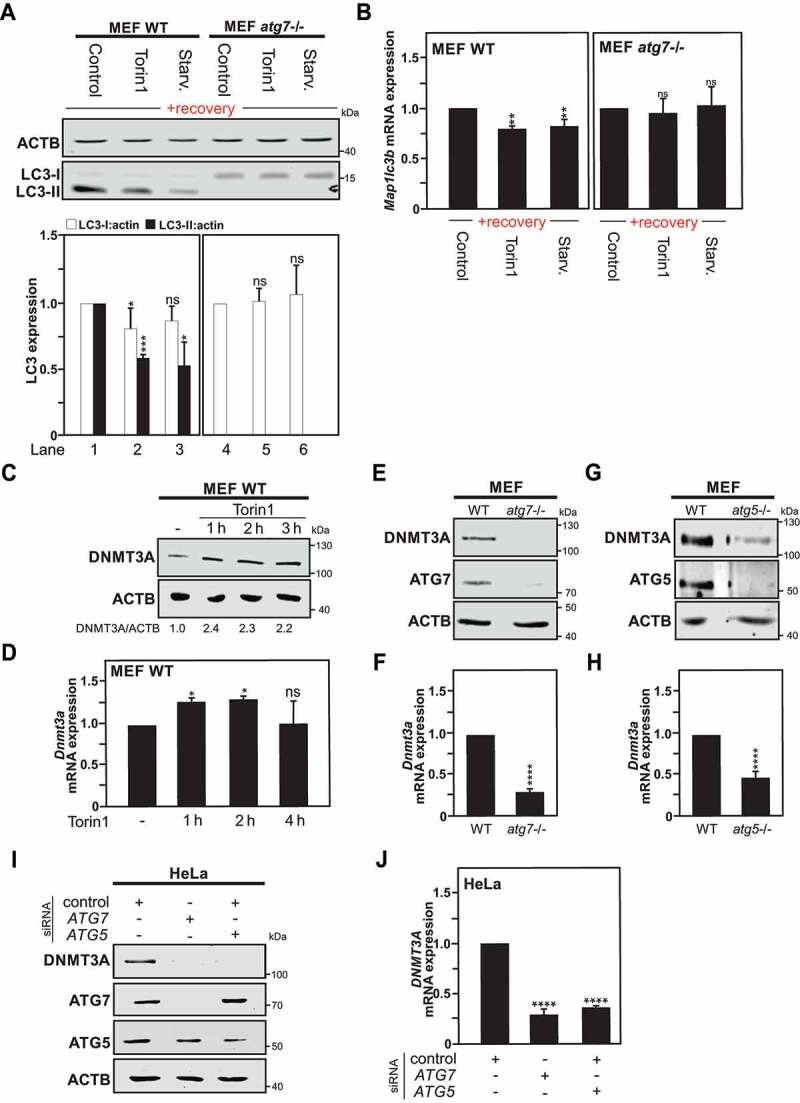
MAP1LC3 downregulation is abrogated in *Atg7*-deficient Cells. (A) Immunoblot analysis of LC3-I and LC3-II expression in Wild type (WT) and *atg7*^−/-^ MEF cells starved (Starv.), treated with torin1, or DMSO (used as control) for 4 h, thereafter left to recover for 1 week. The graphs show the quantification of LC3-I and LC3-II versus ACTB expression. (B) *Map1lc3b* mRNA expression measured by RT-qPCR in previously autophagy-exposed WT and *atg7^−/-^* MEF cells processed as described previously in Fig. S1A. (C) Immunoblot of DNMT3A protein expression in WT MEF cells upon torin1 treatment or DMSO (used as control) at the indicated time points. (D) mRNA expression analysis of *Dnmt3a* level upon torin1 treatment at different time points in WT MEF cells. (E and G) DNMT3A protein expression in *atg7^−/-^* MEF cells (E) or *atg5^−/-^* MEF cells (G) compared to WT MEF cells. (F and H) *Dnmt3a* mRNA expression measured by RT-qPCR in *atg7^−/-^* MEF cells (F) or *atg5^−/-^* MEF cells (H) versus autophagy-proficient cells (WT). (I) Representative immunoblot of 3 independent experiments of DNMT3A, ATG5 and ATG5 expression in HeLa cells after siRNA-mediated *ATG5* or *ATG7* silencing in HeLa cells after 48 h transfection. (J) *DNMT3A* mRNA expression measured by RT-qPCR in HeLa cells treated as described in panel I. All values are means of at least 3 independent experiments ± SEM and considered significant for *p < 0.05, **p < 0.01, ***p < 0.001, ****p < 0.0001. n.s., not significant for the indicated comparison. (n = 3)

*Atg7* gene deficiency in cells is associated with the incapacity to undergo autophagy [36]. ATG7 is a central player for the autophagic process, acting as E1-like activating enzyme facilitating both LC3–phosphatidylethanolamine and ATG12 conjugation, thereby exerting roles during the formation as well as the maturation of the autophagosome [37]. Therefore, ablation of this gene directly abrogates the autophagy pathway. With the purpose of obtaining definite evidence that the observed autophagy long-term signature is indeed a direct consequence of autophagy induction *per se*, we took advantage of *Atg7*-deficient MEF cells, and thus autophagy incompetent cells. *atg7*^−/-^ MEF cells and wild-type MEF cells were exposed for 4 h to starvation or torin1 treatment, and thereafter left to recover under normal culture conditions for one week (as previously described in detail in Fig. S1). After the recovery period, wild-type MEF cells previously exposed to an autophagy stimulus exhibited reduced LC3 protein expression whereas *atg7*^−/-^ MEF cells did not exhibit any reduction in the expression of this protein, as monitored by checking LC3-I levels (Figure 5A). Additionally, *Map1lc3b* mRNA levels under these conditions were also found to be unaffected in the autophagy-deficient *atg7*^−/-^ MEF cells, in contrast to the downregulation of *Map1lc3b* mRNA observed in autophagy-proficient wild-type MEF cells (Figure 5B). Furthermore, the impact of *Atg7* gene deficiency on the regulation of *Dnmt3a* gene expression was also investigated. Induction of autophagy in wild-type MEF cells using torin1 treatment at the indicated time points, led to the upregulation of DNMT3A protein as well as mRNA level, indicating in these mouse cells as in the human ones tested, that autophagy induction is linked to increased *Dnmt3a* gene expression (Figure 5C,D). On the contrary, *Atg7* gene depletion was found to be sufficient in downregulating *Dnmt3a* gene expression, as illustrated by a robust decrease in DNMT3A protein and mRNA levels observed in *atg7*^−/-^ MEF cells, as compared to wild-type MEF cells (Figure 5E,F). To exclude that the observed decreased in DNMT3A expression in *atg7*^−/-^ MEF cells is not due to an ATG7-mediated specific effect, we took advantage of MEF cells deficient for another core protein involved in autophagy, *i.e., atg5*^−/-^ MEFs. In fact, *atg5*^−/-^ MEFs, like *atg7*^−/-^ MEFs, exhibited significantly reduced *Dnmt3a* mRNA, and DNMT3A protein expression levels, when compared with WT MEFs (Figure 5G,H). Pools of siRNA directed against *ATG5* or *ATG7* gene expression where also used to repress the expression of these two ATGs in HeLa cells. Likewise, HeLa cells transfected with either of the siRNA pools targeting *ATG5* or *ATG7* gene expression showed reduced *DNMT3A* mRNA, and DNMT3A protein expression levels, as compared to mock-transfected control cells (Figure 5I,J). Collectively, this data indicates that autophagy-proficiency is required for the acquisition of the described autophagy long-term memory signature.

### Cells previously exposed to an autophagy stimulus are sensitized to an apoptotic stimulus

**Figure 6.**
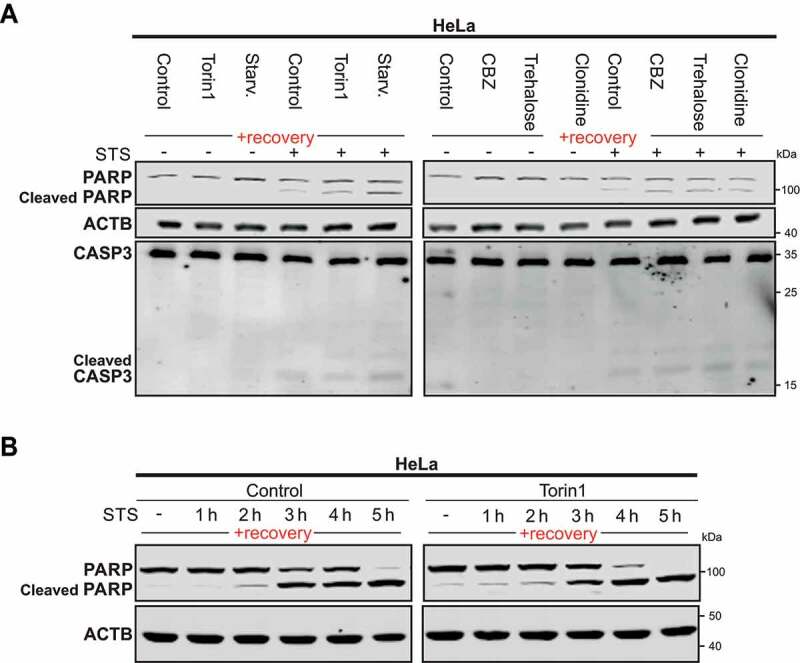
Previously-autophagy exposed cells are sensitized to apoptosis. (A) Representative immunoblot of 3 independent experiments of PARP and CASP3 in Hela cells starved (Starv.), treated with torin1 or MTOR-independent inducers for 4 h, thereafter left to recover for 2 weeks under normal cell culture conditions and treated with staurosporine (STS) for 3 h before harvest. CBZ., carbamazepine. (B) Representative immunoblot of 3 independent experiments of PARP and cleaved-PARP immunoblot of HeLa cells treated for 4 h with DMSO (used as control) and torin1 and left to recover for 2 weeks as previously described in Fig. S1A. Cleaved PARP was analyzed after STS treatment at different time points

We report that exposure of cells to an autophagy stimulus, even for a short period of time, *e.g*., 4 h, and even after a significant recovery period, *e.g*., 1–4 weeks, are associated with sustainable decreases in the expression of *MAP1LC3* genes, which appear to impact on both baseline and stimulus-induced autophagy in these cells (Figure 5). Because interplays between autophagy and apoptosis are reported, we decided to explore whether the second named process is affected in cells previously exposed to an autophagic challenge. Indeed, depending on the cellular context autophagy can exert positive or negative effect on the occurrence of cell death [38]. Therefore, we investigated if HeLa cells previously exposed to MTOR-dependent or MTOR-independent inducers of autophagy would respond differently to staurosporine (STS) induced-apoptotic cell death after 2 weeks of recovery. It appears that, as compared to previously unchallenged cells, cells which have been previously exposed to an autophagy stimulus exhibited higher processing of CASP3 and cleavage of its nuclear substrate PARP upon STS treatment for 3 h, suggesting a sensitization of these cells to this death stimulus (Figure 6A). Furthermore, cells previously exposed to torin1 for 4 h and then left to recover for the same period of time mentioned above, also showed a faster response to the apoptotic stimulus, as illustrated by detection of cleaved PARP at earlier time point upon STS treatment, as compared to control cells (Figure 6B). Altogether, these results show that cells that acquired an autophagy epigenetic memory are more sensitive to an apoptotic stimulus when compared with their parental counterparts. Worth a note, it has been reported that genetic or pharmacological inhibition of autophagy in cancer cells leads to the sensitization of these cells to apoptotic stimuli. These reports support our findings that a decrease of baseline autophagy over time on those previously exposed with an autophagy stimulus appear to have increased susceptibility to cell death [39,40].

### Short autophagy induction leads to a persistent downregulation of MAP1LC3 in vivo

**Figure 7.**
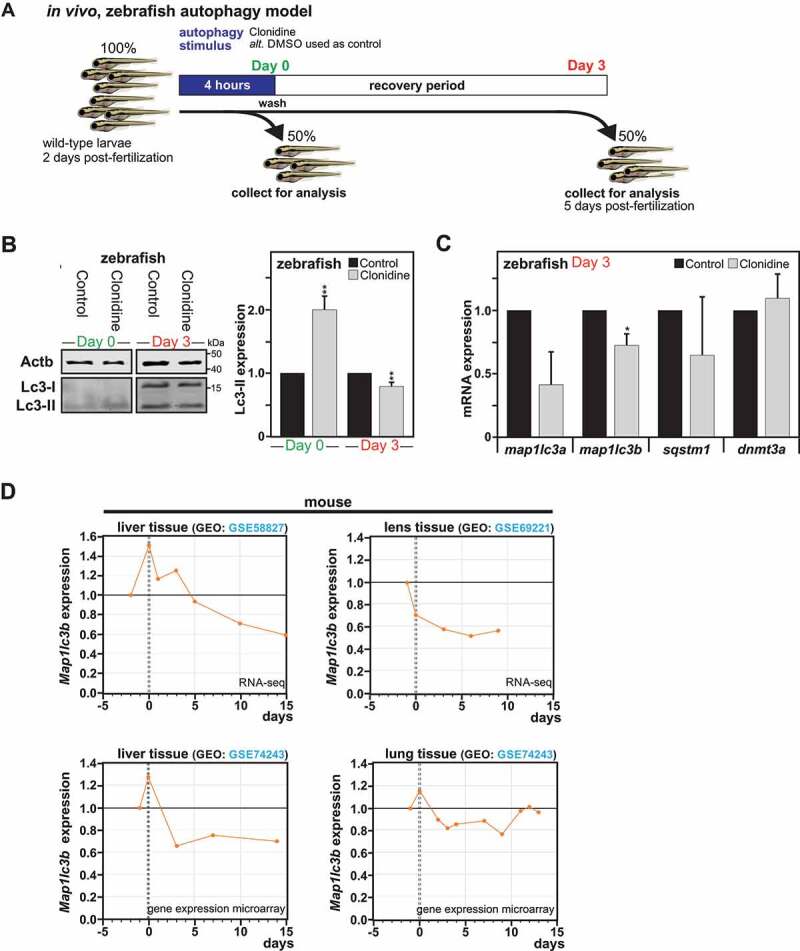
Impact of autophagy on long-term downregulation of MAP1LC3 expression *in vivo*. (A) Illustration of the experimental setting performed in Zebrafish larvae. (B) Representative immunoblot for LC3-II expression in Zebrafish larvae exposed for 4 h to clonidine or DMSO (used as control), collected directly after exposure (Day 0) or left to recover for 3 d. (B) Quantification of LC3-II versus ACTB expression from Zebrafish larvae at day 0 and day 3. All values are means ± SEM (n = 4, with 30 fish per condition). (C) *map1lc3a, map1lc3b, sqstm1* and *dnmt3a* mRNA expression measured by RT-qPCR in zebrafish after the recovery period (n = 3, with 20 fish per condition). (D) Re-analysis of *Map1lc3b* gene expression in different mouse tissues such as liver, lens and lung before and after placenta withdrawal from 4 independent genome-wide data sets, with 0 corresponding to time of birth. All values are a means of at least 3 independent experiments ± SEM and considered significant for *p < 0.05, **p < 0.01, n.s., not significant for the indicated comparison

Finally, to evaluate whether a short autophagy induction can also promote a persistent effect on MAP1LC3 expression *in vivo*, zebrafish (*Danio rerio*), with orthologs to mammalian *DNMT3A*, was used as an animal model to study autophagy in a whole organism [41,42]. Larvae were exposed for 4 h to clonidine, an MTOR-independent autophagy inducer, or DMSO as control. Clonidine was selected based on the previous establishment of a maximum tolerated concentration of this compound at 30 µM in larval zebrafish at 2 d post-fertilization and its broad use and ability at this dose to induce a significant autophagic response in this animal model [43]. After the indicated treatment, larvae were either collected immediately for analysis of the initial autophagic response or left to recover for a 3 d period (Figure 7A). Immunoblot analysis showed an increase of Lc3-II levels directly after autophagy induction at day 0. However, after the recovery period, autophagy pre-treated zebrafish larvae exhibited a significant decrease in Lc3-II expression as compared to the control group (Figure 7B). Analysis of *map1lc3a, map1lc3b, sqstm1*/*p62* and *dnmt3a* mRNA expression levels by RT-qPCR showed significant decreases of *map1lc3b* gene expression after the recovery period. Note that the *map1lc3a* gene expression despite a similar trend as *map1lc3b* did not reach significance (Figure 7C). Altogether these results, confirm the existence of a long-term effect of a short autophagy induction on LC3/Lc3 expression *in vitro* and even in a whole organism.

Moreover, mammals experience periods of starvation throughout life. At birth, following the sudden termination of the trans-placental nutrient supply, some organs suffer temporary but severe starvation, which triggers a transient autophagic response [44,45,46]. The induction of autophagy helps release the amino acids and energy sources necessary for survival until supply can be restored through milk nutrients. In search of a physiological relevance to the phenomenon we uncovered, we took advantage of available genome-wide data sets from four independent studies that investigated the C57/B6 mouse transcriptome in the liver [47,48], lung [49] and lens tissues [50] at different ages covering embryonic stages, right after birth and before the start of suckling, and postnatal developmental stages. Analysis of these data sets revealed in all four studies that *Map1lc3b* gene expression is found to be significantly downregulated at early post-natal stages as compared to just before birth, indicating that in these tissues, the transient induction of autophagy observed directly after birth is associated with a sustained downregulation of *Map1lc3b* gene expression (Figure 7D).

The transcriptomic data available for the murine lung tissues [49] was further exploited to examine if the expression of other autophagy-related genes (*Gabarap, Gabarapl1, Gabarapl2, Atg5, Atg7*, and *Ulk1)*, as well as *Dnmt3a* and *Cdkn2a* genes, are affected by the early neonatal starvation period. The first observation to be made from these transcriptomic analyses, is that at embryonic day 18.5, just before birth, *Map1lc3b, Gabarap and Gabarapl2* genes are expressed in these tissues at significantly higher levels, as compared to the other examined genes (Fig. S7A). Then, it appears that during the early neonatal period of the investigated genes, only *Map1lc3b* exhibited a significant and sustained decrease in gene expression (Note: *Gabarap* gene expression showed significant decrease at postnatal day 9). *Dnmt3a* gene expression showed over time significant increase in gene expression (Fig. S7B). Of importance, autophagy is not reported to occur in the brain tissue as birth, suggesting that this organ is not be affected to the same extend by the withdrawal of the placenta.

Interestingly, human transcriptomic data are available for brain tissues [51] (raw data available at www.humanbraintranscriptome.org and GEO database GSE25219), and the *MAP1LC3B* gene expression levels are not found to be affected in the periods preceding and following birth, indicating that in this particular organ, the overall *MAP1LC3* gene expression was not affected by the developmental stage. Thus, a correlation appears to exist between the observed induction of autophagy observed after the severe starvation following the sudden termination of the trans-placental nutrient supply and a decrease in *Map1lc3b* gene expression in the affected organs. Collectively, these data indicate that the sustained downregulation of *MAP1LC3B* gene expression in response to the transient induction of autophagy can be observed in a physiologically relevant context.

## Discussion

Under physiological conditions, a basal level of autophagy is required to maintain cell homeostasis. However, there is supporting evidence that autophagy activity decline with aging. It has been suggested that this decline might contribute to increased cellular stress and accumulation of damaged organelles that could lead to the development of age-related diseases such as neurodegenerative diseases, cancer, metabolic disorders, and many others [52,53]. Whereas there is physiological *in vivo* evidence that autophagy declines with aging, the mechanism responsible for this process still needs to be elucidated. Here, we uncovered a novel role for *de novo* DNMT3A-mediated DNA methylation in the long-term transcriptional control of *MAP1LC3* gene expression in the context of autophagy induction. Unexpectedly, we found that the partial inhibition of and downregulation of basal MAP1LC3 expression functions as a heritable epigenetic mechanism associated with reduced baseline autophagy.

While previous studies reported the involvement of several histone modifications in the short-term transcriptional regulation of autophagy, epigenetic mechanisms behind possible long-lasting effects of autophagy remained to be elucidated. Therefore, we investigated whether epigenetic changes might offer a molecular explanation for the long-term response to autophagy induction. In order to address the possibility of an autophagy epigenetic memory established by a previous autophagic stimulus, cells were treated for a short period with MTOR-dependent or – independent autophagy inducers and thereafter left to recover for a prolonged period. Hence, following the recovery period, induced changes must be heritable through each cellular division. As aforementioned, DNA methyltransferase DNMT3A is responsible for *de novo* establishment of DNA methylation patterns of specific genomic regions with the potential of controlling long-lasting effects. For instance, *in vivo* DNA methylation by DNMT3A is required for long-term differentiation of hematopoietic stem cells. Based on our findings, autophagy induction promotes a substantial DNMT3A upregulation, which results in its recruitment to *MAP1LC3A, MAP1LC3B* and *MAP1LC3B2* loci and eventually leads to their transcriptional repression that it is abrogated in *Atg7*-deficient cells due to the lack of DNMT3A expression. Of note, using a gene array, 84 autophagy-associated genes were evaluated for changes in their expression pattern in previously autophagy-exposed cells. Surprisingly, while most tested genes exhibited an unaltered gene expression, the *MAP1LC3* isoforms showed a selective and persistent transcriptional downregulation. This downregulation of MAP1LC3 expression was further confirmed at the protein level. Collectively, those data suggest targeted recruitment of DNMT3A and resulting DNA methylation on the *MAP1LC3* loci, upon autophagy induction.

DNA methylation occurs in a nonrandom manner by generating a specific pattern of gene methylation on CpG sequences *in* vitro and *in vivo*. Therefore, further studies will be required to elucidate the mechanism that guides DNMT3A in targeting these specific genomic loci and the involvement of other factors in this long-term regulation of autophagy. During the past decades, the understanding of the role of DNMTs dynamics in DNA methylation has evolved dramatically. Indeed, there is an increasing number of studies that support the fact that DNA methylation can be mediated by different DNMT-including complexes, histone-modifying enzymes (including polycomb proteins and histone deacetylase enzymes), as well as numerous transcription factors that might contribute to the DNMT-selective silencing of a specific genomic sequence. However, the detailed mechanism explaining how specific *de novo* methylation occurs are still to be fully uncovered [54,55,56,57,58,59,60].

Our findings might have significant physiological implications but also in respect to evolution. A memory of autophagy as a response to starvation, a natural inducer of autophagy, could prepare a cell population or whole organisms for an environment where resources are scarce. Reducing the initial magnitude of autophagy induction allows a prolonged and maintained response, which could allow cells or whole organisms to cope with prolonged withdrawal of nutrients and contribute to the life span extension attributed to autophagy. Moreover, the mechanism that we uncover has a physiological relevance right after birth by the termination of the trans-placental nutrient supply, where some organs such as lung, lens or liver suffer a high starvation period that leads to a decrease of *Map1lc3b* gene expression after postnatal stages until the start of suckling. Additionally, the mechanism that we described brings a better understanding of the molecular mechanism responsible for the decline of autophagy activity during aging, which might lead to the onset of various human disorders, including metabolic conditions, neurodegenerative diseases, cancers and infectious diseases. Recent studies show a strong association between DNA methylation and aging [61]. For instance, an increase in methylation of the *Map1lc3b* promoter region as well as a decrease in its expression was significantly reduced in aged macrophages derived from 62–64 weeks, as compared to their young 8 weeks old counterparts [62].

Given these observations, pharmacological approaches to regulate this pathway are receiving considerable attention [63]. However, here we highlight a possible limitation that the activators of autophagy may encounter as therapeutic drugs. The mechanism that we describe on how cells acquired an epigenetic memory to autophagy upon an initial stimulus could contribute to reduced efficacy of these drugs and could explain acquired drug resistance. In fact, those cells adapt to the previous autophagy stimulus and are still able to undergo autophagy after a second stimulation, however, the amplitude of the induction is decreased due to reduced baseline autophagy. This might lead to the development of tumor resistance to a prolonged treatment with chemotherapy based on autophagy activation.

In summary, this study establishes a novel molecular explanation based on DNA methylation for an acquired epigenetic memory of autophagy, which has the potential of influencing cells or even whole organisms over time and the response to further autophagy-related stimuli.

## Material and Methods

### Reagents and antibodies

Reagents used in this study include bafilomycin A_1_ (BafA1) (Santa Cruz Biotechnology, sc-201,550), carbamazepine (CBZ) (Sigma-Aldrich, C4024), clonidine (Sigma-Aldrich, C7898), Hoechst 33,342 (Molecular probes/Invitrogen, H3570), rapamycin (LC Laboratories, R5000), torin1 (Tocris, 4247), trehalose (Sigma-Aldrich, T9531), staurosporine (STS) (Sigma-Aldrich, S6942), 5-aza-2´-deoxycytidine (5-AZA-CdR) (Sigma-Aldrich, A3656) and ProLong^TM^ Gold antifade Mounting medium with DAPI (Molecular probes/Invitrogen, P36931).

Primary antibodies used in this study include ACTB (mouse monoclonal antibody [mAb]; Sigma Aldrich, A-3853), ATG5 (mouse mAb; Bio-techne, 603,813), ATG7 (rabbit polyclonal antibody [pAb]; Genetex, GTX61647), CASP3 (rabbit mAb; Cell Signaling Technology, 9662), DNMT1 (mouse mAb; Abcam, ab13537), DNMT3A (rabbit pAb; Abcam, ab2850 and Santa Cruz Biotech., sc20703), DNMT3B (mouse mAb; Abcam, ab13604), GAPDH (rabbit pAb; Trevigen, 2275), H3 C-terminal (rabbit pAb; Active Motif, 39,164), LMNB1 (rabbit pAb; Abcam, ab16048), LC3B (rabbit pAb; Sigma Life Science, L7543), PARP1 (rabbit mAb; Cell Signaling Technology, 9532), and SQSTM1/p62 (rabbit pAb; Cell Signaling Technology, 5114).

### Cell culture and transfection

Human non-small-cell lung carcinoma U1810 cells [64] were cultured in RPMI medium 1640 (Gibco, 11,875–093) and transfected using X-tremeGENE™ HP DNA transfection reagent (Roche, 06 366 236 001). Human cervical carcinoma HeLa cells (ATCC® CCL-2™) were freshly obtained from Sigma and were grown with minimum essential medium (MEM) (Gibco, 21,090–022) supplemented with 1% sodium pyruvate (Gibco, 11,360–070) and 1% MEM non-essential amino acids (Gibco, 11,140–050).

Transfections for HeLa Cells were performed using lipofectamine® LTX with PLUS™ reagent or Lipofectamine 3000 (Invitrogen, L3000015). For gene silencing by siRNA, Non-targeting control, DNMT3A ON-TARGET plus SMARTpools siRNAs, whose sequences can be found in **Table S1**, were obtained from Dharmacon. The medium was removed after 3 h of transfection, replaced with fresh medium and harvested after 48 h transfection before BafA1 (Sigma-Aldrich, 19–1489) treatment. For DNMT3A overexpression, HeLa cells were transfected with 2 µg of pcDNA3/Myc-DNMT3A. The latter plasmid was a gift from Arthur Riggs (Addgene, 35,521; http://n2t.net/addgene:35521) or a pcDNA3 empty vector (Invitrogen, V790-20) (control) respectively. The medium was changed after 3 h transfection and harvested after 24 h before following treatment. Both overexpression of the DNMT3A protein and siRNA-mediated knockdowns were confirmed by immunoblotting by comparison to their respective control samples. Mouse embryonic fibroblast (MEF; ATCC® CRL-2214™) cells were cultured with Dulbecco’s modified Eagle’s medium (DMEM) (Gibco, 61,965–026). All mediums were supplemented with 10% fetal bovine serum (Gibco, 10,270–106), 1% L-Glutamine (Gibco, 25,030–024) and 1% penicillin-streptomycin (Gibco, 10,478–016). Cells were cultivated under standard conditions.

### Generation of “previously autophagy-exposed cells” and treatments

U1810, HeLa or MEF cells were seeded at 75,000 cells per well in 6-well plates in complete culture medium (as described above). One-day post-seeding, culture medium was removed, cells were washed twice with PBS (Gibco, SH300028.02) and treated with either DMSO ([Sigma-Aldrich, D4540]; 1:4000, used as control) or one of the following autophagy inducers: 250 nM torin1, 50 μM carbamazepine, 100 mM trehalose or starved using Earle’s Balanced Salt Solution (EBSS medium; Gibco, 24,010–043). After 4 h, cells were collected and transferred into T75 flasks with fresh complete cell culture medium. Thereafter, cells were cultivated for a period of time, ranging from 1 to 4 weeks before experiments were performed. Cells were split 3 times per week during this recovery period. Before performing the experiments, cell number was normalized. An illustrated description of the method is shown in Fig. S1A.

### Immunoblotting

Total protein extracts were made directly in 5x Laemmli buffer (62.5 mM Tris-HCl, pH 6.8, 2% SDS, 10% glycerol (Sigma-Aldrich, G5516), 5% beta-mercaptoethanol (Sigma-Aldrich, M3148), 0.02% bromophenol blue [Biorad, 161–04021]) by scraping of the cells. For immunoblot analysis, protein extracts were sonicated, boiled, resolved by 10% or 15% SDS–polyacrylamide gel electrophoresis and blotted onto 0.2-μm pore-size nitrocellulose membranes (Corning, 431,222) in a wet transfer system (Bio-Rad, Hercules, CA, USA). Membranes were blocked with 0.1% Tween 20 (Sigma-Aldrich, P1379) and 5% milk (Semper, 31,909) in PBS (AH Diagnostics, sc-362,299) and incubated overnight at 4°C with indicated primary antibodies (with details listed above) following manufacturer’s instructions. The day after membranes were incubated with the appropriate anti-rabbit or mouse horseradish peroxidase secondary antibody (1:5000; Pierce, 31,460 and 31,430 respectively) or RDye® 680RD goat anti-rabbit or RDye® 800RD goat anti-mouse IgG secondary antibody (1:5000; LI-COR Bioscience, 926–68,071 and 926–32,210 respectively) for 1 h at room temperature. Immunoblot with anti-ACTB (actin beta) or anti-GAPDH (glyceraldehyde-3-phosphate dehydrogenase) antibodies were used for standardization of protein loading. Bands were visualized either by enhanced chemiluminescence (ECL-Plus; Pierce, 32–132) or using an Odyssey CLx infrared imaging system (LI-COR Biosciences, Lincoln, NE, USA). All targeted proteins of interest were normalized to the selected housekeeping gene, intensity of the bands were verified within the same linear range and quantification was performed using the ImageJ software.

### Subcellular fractionation

Cytoplasmic and nuclear protein fractions were collected using the NE-PER™ Nuclear and Cytoplasmic Extraction Reagents (Thermo Fisher Scientific, 78,835) following the manufacturer´s instructions. After subcellular fraction purification, the samples were resolved by SDS-PAGE and analyzed by immunoblotting as previously described.

### Isolation and culture of mouse primary cortical neurons

All studies and procedures were performed under the jurisdiction of appropriate Home Office Project and Personal animal licenses and with the local Ethics Committee approval (University of Cambridge, UK). Primary cortical neurons were isolated from C57BL/6 mouse embryos at E16.5 (Jackson Laboratories, 000664). Briefly, brains were harvested and placed in ice-cold PBS-glucose (Thermo Fisher, A2494001), where the meninges were removed and the cerebral cortices were dissected. After mechanical dissociation using sterile micropipette tips, dissociated neurons were resuspended in PBS-glucose and collected by centrifugation. Viable cells were cultured in Neurobasal medium supplemented with 2 mM glutamine (Thermo Fisher, 25,030.024), 200 mM B27 supplement (Thermo Fisher, 12,587,010) and 1% penicillin-streptomycin (Thermo Fisher, 15,140,148) at 37°C in a humidified incubator with 5% CO_2_. One half of the culturing medium was changed once three days after seeding. After 6 d of culturing *in vitro*, differentiated cortical neurons were treated as indicated for 4 h, after which cell culture medium was replaced by fresh medium. Thereafter, neurons were cultured for an additional 8 d period, changing half of the medium every 3 d. For the assessment of autophagy by LC3-II levels, a saturating concentration of 400 nM BafA1 was added to the cells in the last 4 h before harvesting.

### Collection and maintenance of zebrafish embryos

Zebrafish (*Danio rerio*) embryos of AB strain were obtained from the Zebrafish core facility at Comparative Medicine, Karolinska Institutet (Stockholm, Sweden). Zebrafish larvae were collected and staged according to the established criteria [65]. Embryos were raised under standard conditions on 14 h light-dark photoperiod and reared in zebrafish embryo medium, EM (5 mM NaCl, 0.17 mM KCl, 0.33 mM CaCl2, 0.33 mM Mg_2_SO_4_, 5 mM HEPES (Sigma-Aldrich, H3375), pH 7.2) [43].

### Compound exposure experiments in larval zebrafish

Compound exposure experiments were performed on wild-type larvae from 2 to 5 days post-fertilization (dpf) in the dark at 28°C. The maximum tolerated concentration of 30 μM clonidine was previously determined [43]. The treatment with clonidine was performed on enclosed larvae at 2 dpf (n = 60 per condition) for 4 h. After the initial stimuli, 30 larvae were collected immediately for analysis and the other half were washed three times in fresh EM and collected at 5 dpf. EM was replaced daily. After the indicated treatment, larvae were transferred to prechilled tubes with EM and the yolk was burst. Each sample was homogenized with 1x IPSC7 supplemented with HaltTM Protease and Phosphatase Single-use Inhibitor Cocktail (Thermo Fisher Scientific, 78,442) and PMSF (Sigma-Aldrich, P7626) and snap-frozen into cryotubes. Samples were then subjected to either immunoblotting analysis or RNA extraction. For immunoblotting analysis, samples were quantified and 40 μg of protein were further processed and analyzed by immunoblotting as previously described. Total RNA isolation was performed following the manufacturer’s instructions of ReliaPrep RNA tissue Miniprep System (Promega, Z6010). Samples were lysed in lysis buffer (LBA; Promega, Z6112). Two spatulas of Zirconium Oxide Beads 0.5 mm diameter, RNase Free (Next Advance, ZROB05) were added to each sample and homogenized using a bullet blender (Next Advance; speed 6 for 3 min). The rest of the RNA isolation was continued according to the instructions. RNA quantifications of the different samples were determined using a NanoDrop ONE® spectrophotometer (Thermo Fisher Scientific, Wilmington, DE, USA). cDNA was synthesized from 500 ng of RNA using iScript cDNA synthesis (Bio-Rad, 1,708,890). qPCR was run on a StepOnePlus Real-Time PCR system (Applied Biosystems, Foster city, CA, USA) using Fast SYBR™ Green Master Mix (Qiagen, 4,385,610). *Actb1* was used as a housekeeping gene. List of primers can be found in **Table S2**. Statistical analysis was performed using R.

### Immunofluorescence and confocal microscopy

For confocal microscopy analysis, the adherent mammalian cells were grown on coverslips for 24 h. 4% Paraformaldehyde-fixed cells were blocked in HEPES 10 mM, 3% bovine serum albumin (Sigma-Aldrich, A9418), 0.3% Triton X-100 (Sigma-Aldrich, T8787) diluted in PBS (1 h at room temperature) and incubated with the indicated primary (4°C, overnight) and secondary AlexaFluor® 488 goat anti-rabbit IgGs (1:1000; Molecular probes/Invitrogen, A11008; room temperature, 1 h) antibodies. Antibodies are listed in above section. Nuclei were counterstained with Hoechst 33,342 (Molecular Probes/Invitrogen, H3570). Samples were mounted with Vectashield® Antifade Mounting Medium (Vector Laboratories, H-1900) and analyzed with Zeiss LSM700 confocal laser scanning microscopy (Oberkochen, Germany).

### Electron microscopy

Electron microscopy was performed on HeLa and MEF cells after 2 and 1 week recovery periods, respectively. After this period, cells were seeded on 6-well plates and the day after the cells were washed twice with 1xPBS and left with 0.25% trypsin for 5 min. Thereafter, the cells were washed with PBS twice with gentle centrifugation, and the supernatant was then removed and resuspended with 1 mL of fixative solution (2.5% glutaraldehyde in 0.1 M phosphate buffer, pH 7.4) for 1 h at room temperature and then store at 4°C before processing. The fixed cells were rinsed in 0.1 M phosphate buffer, pH 7.4 and post-fixed in 2% osmium tetroxide 0.1 M phosphate buffer, pH 7.4 at 4°C for 2 h, followed by stepwise ethanol and acetone dehydration and finally embedded in LX-112 (Ladd, 21,210 – LX 112). Ultrathin sections (approximately 60–80 nm) were prepared using a Leica EM UC 7 ultramicrotome (Leica, Vienna, Austria) and contrasted with uranyl acetate followed by lead citrate and imaged in a Hitachi HT7700 transmission electron microscope (Hitachi Hightech, Japan) at 100 kV. Digital images were acquired using a 2 kx2k Veleta CCD camera (Olympus Soft Imaging Solutions, GmbH, Münster, Germany).

### FACS analysis

Fluorescence associated cell sorting was performed using Alexa Fluor® 488 Annexin V/Dead Cell apoptosis Kit (Life Technologies, V13241) according to manufacturer’s instructions. The data were acquired on a Novocyte flow cytometer (ACEA Biosciences, San Diego, CA, USA) and analyzed with FlowJo version 10.1r5 software (TreeStar Inc, Ashland, ORE, USA).

### RNA isolation, cDNA synthesis, and qPCR

Total RNA was extracted using the RNeasy Mini Kit (Qiagen, 74,104), including on-column DNase digestion. RNA concentrations were determined using the NanoDrop® spectrophotometer (Thermo Fisher Scientific, Wilmington, DE, USA). cDNA was synthesized from 1 μg RNA using oligo dT, dNTPs, and Superscript II or III Reverse Transcriptase (Invitrogen, 18,064,022 and 18,080,044). qPCR was run on an ABI 7500 (Applied Biosystems, Foster City, CA, USA) using the SYBR™ Green master mix (Qiagen, 4,385,610). GAPDH and B2M were used as housekeeping genes for normalization. The specific primer sequences are listed in **Table S2**. Results were calculated using the delta Ct method and represented as a fold over untreated cells. Analysis and statistical evaluation were done using R data analysis software.

### 5-Aza-2ʹ-deoxycytidine (5-AZA-CdR) treatment

HeLa cells (100,000 per well) were seeded in 6-well plates. The day after cells were treated with 1 or 10 µM of 5-Aza-2´-deoxycytidine (Sigma Aldrich, A3656) or DMSO as control for 48 h. Cells were then harvested either for immunoblotting or for RNA extraction as previously described.

### Gene expression array analysis

The human autophagy RT2 profiler PCR array (Qiagen, PAHS-084ZE) was used to profile the expression of 84 genes central to the autophagy process using manufacturer’s instructions. cDNAs were synthesized from 1 μg of mRNA using the RT2 First Strand Kit from Qiagen. qPCR was run on an ABI 7900 HT (Applied Biosystems, Foster City, CA, USA) in a 384 well format using the Fast SYBR™ Green master mix (Qiagen, 4,385,610). Data were analyzed using the Qiagen online tool.

### DNA extraction and DNA methylation analysis

DNA extraction was performed using the QIAamp DNA Mini Kit (Qiagen, 51,304). DNA concentrations were measured using the Qubit™ fluorometer (Thermo Fisher Scientific, London, United Kingdom). Whole-genome DNA methylation analysis was done using the Illumina® Infinium HumanMethylation450 BeadChip array (San Diego, CA, USA). Experiments were performed according to protocol at the Bioinformatics and Expression Analysis (BEA) Core facility at Novum, Karolinska Institutet.

### Chromatin immunoprecipitation (ChIP)

DNMT3A and DNMT3B ChIP experiments were done using the HighCell# ChIP kit (Diagenode, kch-mahigh-G48) according to the manufacturer´s instructions. 3 million cells (HeLa and U1810) were seeded in 10 cm Petri dishes and treated with torin1 or DMSO at indicated timepoints. Briefly, after cell cross-linking in 1% formaldehyde and cell lysis, chromatin shearing was done with a Bioruptor® Pico sonicator (Diagenode, Liège, Belgium). Then, each chromatin immunoprecipitation was done using 6.3 μg of antibody (Listed in section above) overnight at 4°C, with normal rabbit IgGs (R&D systems, AB-105-C) used as a control. Purified DNA and 1% input were then analyzed by qPCR (primers sequences are listed in **Table S2**). Data interpretation from qPCR was done by calculation of the percentage to input and then normalized to the control condition.

### Analysis of methylated DNA by MS-qPCR

Cells (100,000) were harvested after treatment with DMSO used as control or torin1 for 24 h in HeLa cells that were previously transfected for 48 h either with siRNA Control or siRNAs against *DNMT3A*. Lysis and bisulfite conversion were carried out using the EpiTect Fast lyseAll Bisulfite kit (Qiagen, 59,864) and following the manufacturer´s instructions. Converted DNA (1 µL) was used for PCR using the HotStarTaq Master Mix (Qiagen, 203,443). PCR was run in a T100™ Thermal Cycler (Bio-Rad, Hercules, CA, USA) at 56°C and 34 cycles using the corresponding MS-PCR primers listed in **Table S2**. Design of the MS-PCR primers was determined using the online tool Methprimer. The PCR products from the Bisulfite converted samples were run on a 2% agarose gel containing Syber safe (Invitrogen, S33102) and revealed using a Gel Doc™ EZ Imager (Bio-Rad, Hercules, CA, USA).

### GRO-Sequencing

GRO-Seq experiments were performed as previously reported [8,66]. Briefly, cells were washed with cold 1X PBS buffer and swelled in swelling buffer (10 mM Tris-Cl, pH 7.5, 2 mM MgCl_2_, 3 mM CaCl_2_) for 5 min on ice and harvested. Cells were lysed in lysis buffer (swelling buffer with 0.5% IGEPAL 630 (Sigma-Aldrich, I8896), 2 u/ml SUPERase In [Invitrogen, AM2694] and 10% glycerol) and finally resuspended in 100 μL of freezing buffer (50 mM Tris-Cl pH 8.3, 40% glycerol, 5 mM MgCl2, 0.1 mM EDTA). For the run-on assay, resuspended nuclei were mixed with an equal volume of reaction buffer (10 mM Tris-Cl, pH 8.0, 5 mM MgCl2, 1 mM DTT, 300 mM KCl, 20 units of SUPERase In, 1% sarkosyl [Sigma-Aldrich, L7414], 500 μM ATP, GTP, and Br-UTP [Sigma-Aldrich, B7166], 2 μM CTP [Affimetrix, #77,245]) and incubated for 5 min at 30°C. The nuclear-run-on RNA (NRO-RNA) was then extracted with TRIzol LS reagent (Invitrogen, 10,296,028) following the manufacturer’s instructions. NRO-RNA was then subjected to base hydrolysis on ice for 40 min and followed by treatment with DNase I and Antarctic phosphatase (New England Biolabs/NEB, M0303 and M0289). To purify the Br-UTP-labeled nascent RNA, the NRO-RNA was immunoprecipitated with anti-BrdU agarose beads (Santa Cruz Biotechnology, sc-32,323 AC) in binding buffer (0.5X SSPE [Sigma-Aldrich, S2015], 1 mM EDTA, 0.05% Tween-20 [Sigma-Aldrich, P1379]). To repair the end, the immunoprecipitated BrdU-RNA was resuspended in a 50-μL reaction (45 μl DEPC water [Invitrogen, 10,977–035], 5.2 μl T4 PNK buffer, 1 μl SUPERase In and 1 μl T4 PNK [New England Biolabs/NEB, M0236]) and incubated at 37°C for 1 h. The RNA was extracted and precipitated using acidic phenol-chloroform. The cDNA synthesis was basically performed with a few minor modifications as described in [67]. First, RNA fragments were subjected to a poly-A tailing reaction by poly-A polymerase (NEB, M0276) for 30 min at 37°C. Subsequently, reverse transcription was performed using oNTI223 primer (5ʹ-pGATCGTCGGACTGTAGAACTCT;CAAGCAGAAGACGGCATACGATTTTTTTTTTTTTTTTTTTTVN). Second, tailed RNA (8.0 μL) was subjected to reverse transcription using superscript III (Invitrogen, 18,080,093). The cDNA products were separated on a 10% polyacrylamide TBE-urea (Sigma-Aldrich, U5378) gel and the extended first-strand product (100–500 bp) was excised and recovered by gel extraction. After that, the first-strand cDNA was circularized with CircLigase (Epicenter, CL4111K) and relinearized using Ape1 (NEB, M0282). Relinearized single strand cDNA (sscDNA) was separated on a 10% polyacrylamide TBE gel and the product of needed size was excised (~120-320 bp) for gel extraction. Finally, sscDNA template was amplified by PCR using the Phusion High-Fidelity enzyme (NEB, M0530) according to the manufacturer’s instructions with two oligonucleotide primers: oNTI200: (5ʹ-CAAGCAGAAGACGGCATA) and oNTI201 (5ʹ-AATGATACGGCGACCACCGACAGGTTCAGAGTTCTACAGTCCGACG). DNA was then sequenced on the Illumina HiSeq2000 according to the manufacturer’s instructions, using the small RNA sequencing primer 5ʹ-CGACAGGTTCAGAGTTCTACAGTCCGACGATC.

### Statistical analyses

Statistical differences were performed using the Student’s t-test; *p < 0.05, ** p < 0.01, *** p < 0.001, **** p < 0.0001. n.s., not significant for indicated comparison. Error bars indicate standard error mean (SEM) of at least three independent experiments.

## Supplementary Material

Supplemental Material

Supplemental Material
