# Supplementary material for: The DNA methyltransferase DNMT3A contributes to autophagy long-term memory
Source: Autophagy. 2020 Sep 14;17(5):1259–77. doi: 10.1080/15548627.2020.1816664 (PMC8143216; doi:10.1080/15548627.2020.1816664)
Supplement: Supplemental Material [file KAUP_A_1816664_SM0486.docx]

**Supplemental material**

**
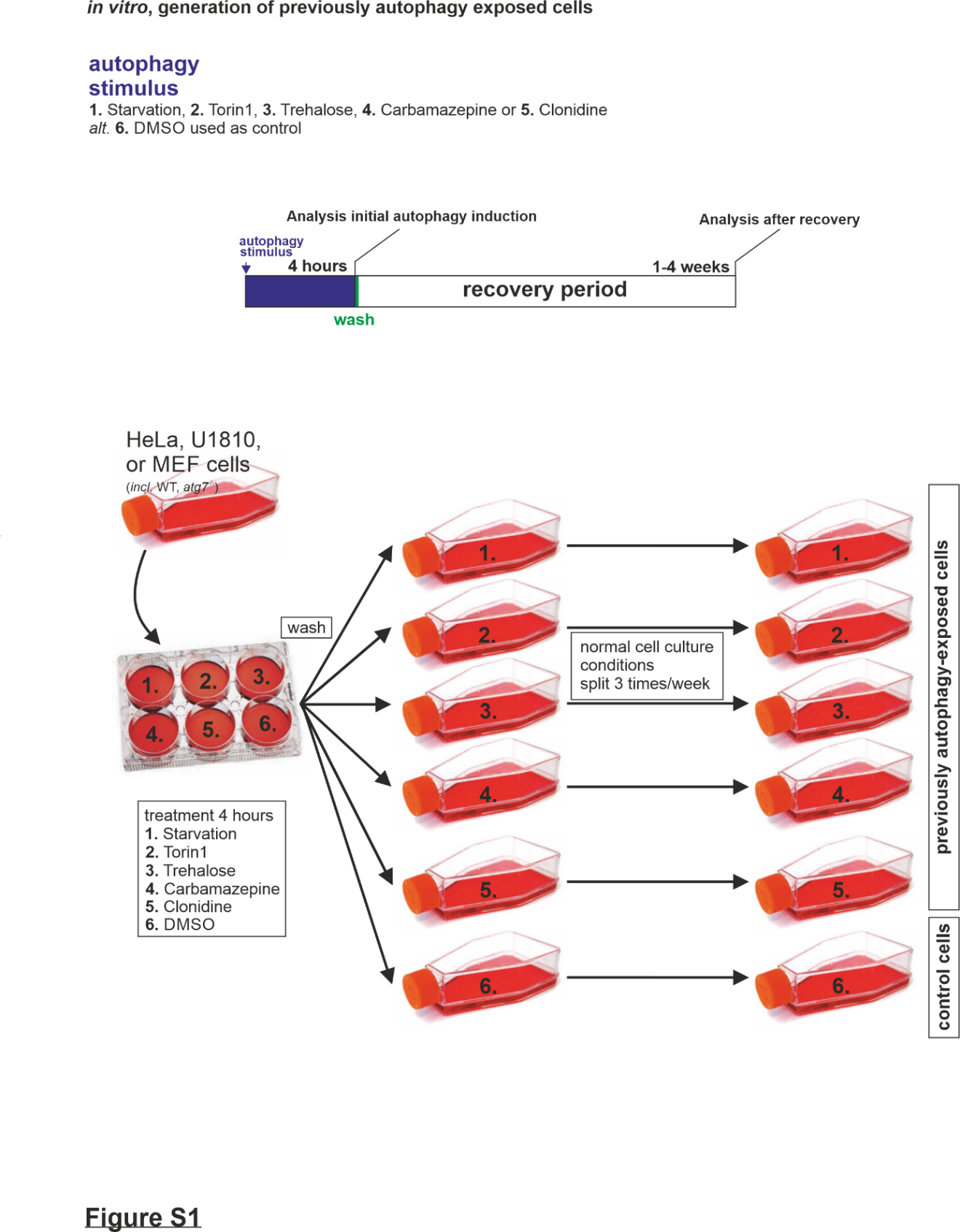
**

**Figure S1.** Detailed illustration of the *in vitro* model used to generate previously autophagy-exposed cells. Schematic diagram of how “previously autophagy-exposed cells” were generated.

**
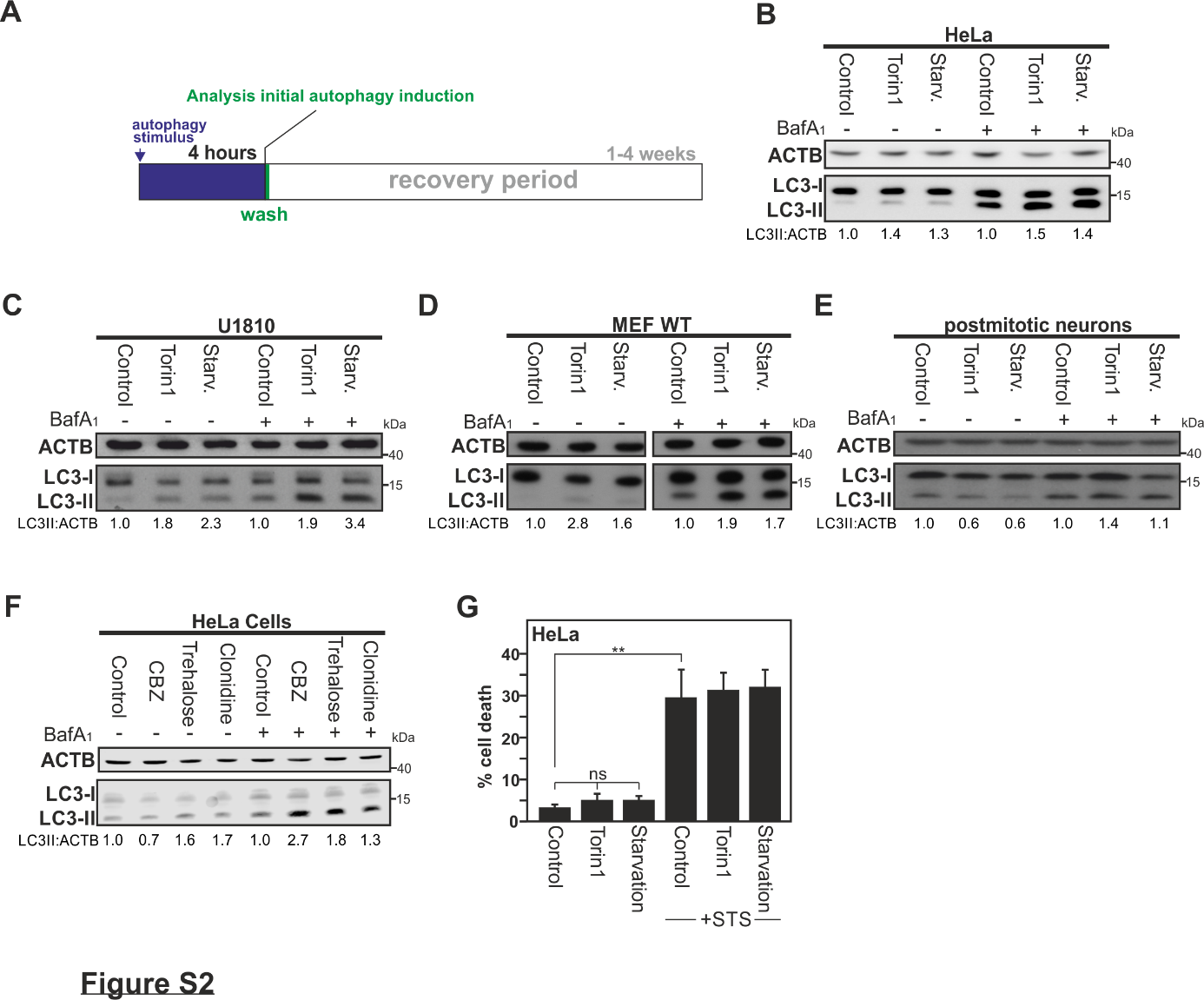
**

**Figure S2**. Characterization of initial cell response to autophagic stimuli. (**A**) Schematic illustration that indicates that the analysis performed in the following panels corresponds to 4 h autophagy stimulus. (**B** to **F**) HeLa cells (**B**), U1810 cells (**C**), MEF cells (**D**), postmitotic neurons (**E**) exposed to autophagic stimuli, either with mTOR-dependent inducers (starvation [Starv.] or torin1) or (**F**) MTOR-independent inducers (carbamazepine [CBZ], trehalose or clonidine) for 4 h. Bafilomycin A_1_ (BafA1; 40 nM or 400 nM for postmitotic neurons) was used as a co-treatment when indicated. LC3 immunoblot analysis revealed that the initial treatment with autophagy inducers was linked to the occurrence of autophagy, as established by an increased lipidation of LC3. (**G**) HeLa cells were exposed to autophagic stimuli, starvation using EBSS or 250 nM torin1 treatment for 4 h. Thereafter, cell death was analyzed by flow cytometry using the FITC-ANXA5/Annexin-propidium iodide staining. Co-treatment with 1 µM staurosporine (STS) was used as an inducer of cell death. Representative data of 3 independent experiments are shown. In panel G, values are means of 3 independent experiments ± SEM and considered significant for **p<0.01, n.s., not significant for the indicated comparison.


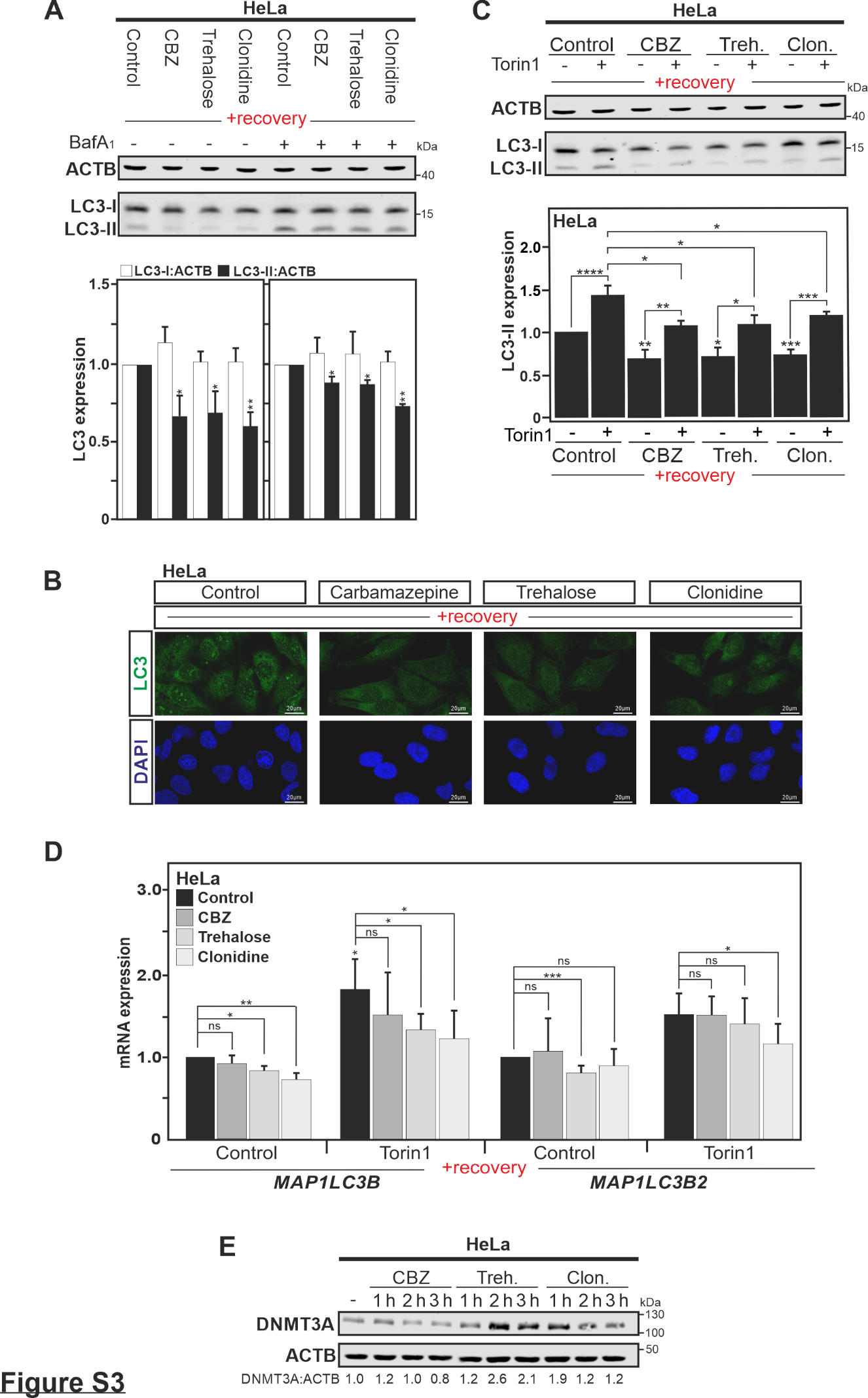


**Figure S3.** Initial induction by MTOR-independent inducers of autophagy promotes a similar downregulation of MAP1LC3B expression after recovery. (**A**) HeLa cells were exposed to 50 µM carbamazepine (CBZ), 100 mM trehalose (Treh.) or 40 µM clonidine (Clon.) treatment for 4 h. Downregulation of LC3 expression was observed after a two weeks recovery period as observed by LC3 immunoblot of autophagy pretreated cells compared to controls. Pre-treatment with 40 nM bafilomycin A_1_ (BafA1) before harvesting the samples, showed that the observed decrease in LC3 expression was not the result of an increase in autophagic flux. Representation of at least 3 independent experiments. The graph is shown as mean ± SEM of the quantification of LC3-I and LC3-II versus ACTB expression. (**B**) Immunofluorescence confocal microscopy staining for endogenous LC3 in HeLa cells after recovery for 2 weeks. (**C**) Immunoblot and quantification analysis of LC3-II expression in HeLa cells, previously exposed to an autophagy stimulus upon re-stimulation of autophagy with torin1 treatment for 1 h (+) as compared with the control ones (-). (**D**) *MAP1LC3B* and *MAP1LC3B2* isoforms mRNA expression measured by RT-qPCR in previously autophagy-exposed cells as compared to their parental untreated HeLa cells counterparts at baseline levels after recovery and upon re-stimulation of autophagy with torin1. (**E**) Representative immunoblot of DNMT3A versus ACTB expression upon CBZ, trehalose or clonidine at indicated time points, compared with the control one, treated with DMSO (n=4-8). All values are means of at least 3 independent experiments ± SEM and considered significant for *p<0.05, **p<0.01, ***p<0.001, ****p<0.0001. n.s., not significant for the indicated comparison.

**
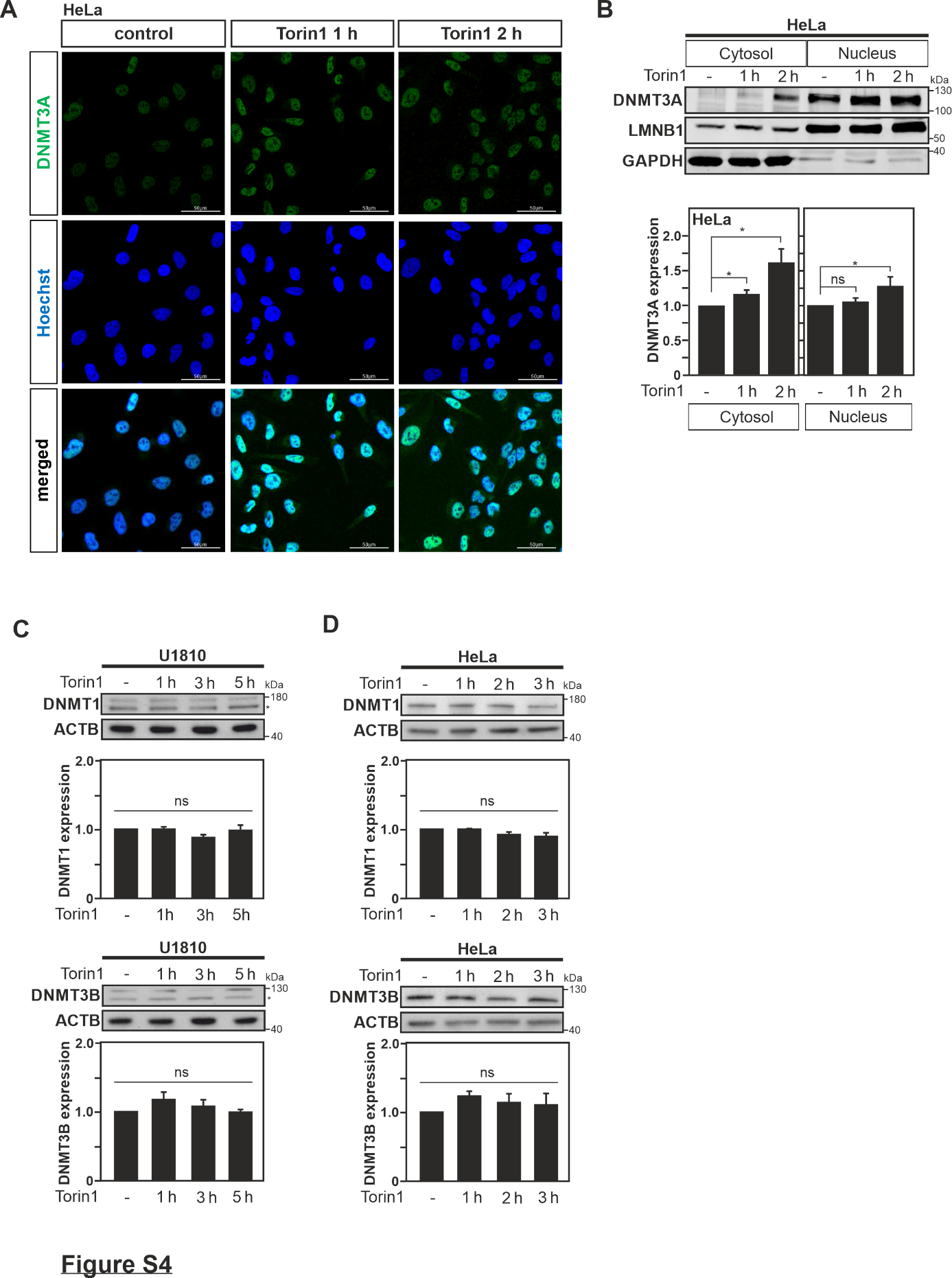
**

**Figure S4**. Investigation of DNMT3A subcellular localization and DNMT1 and DNMT3B protein expression upon torin1 induced autophagy. (**A**) DNMT3A expression and cellular localization were analyzed by confocal immunofluorescence using an immunostaining for DNMT3A and a Hoechst nuclear counterstain upon autophagy induction with 250 nM torin1 at indicated time points. (**B**) DNMT3A, LMNB1 and GAPDH expression in cytosolic versus nuclear subcellular fractions analyzed by immunoblot analysis. Quantification and statistical analysis of DNMT3A subcellular localization from 3 independent experiments are presented in the graph. (**C**) DNMT3B and DNMT1 immunoblot analysis in HeLa and U1810 cells treated with torin1 at indicated time points. *, refers to the indicated protein. Quantification and statistical analysis of DNMT3B and DNMT1 expression from 3 independent experiments are presented in the graphs below the corresponding immunoblots. All values are a mean of at least 3 independent experiments ± SEM and considered significant for *p<0.05. n.s., not significant for the indicated comparison.


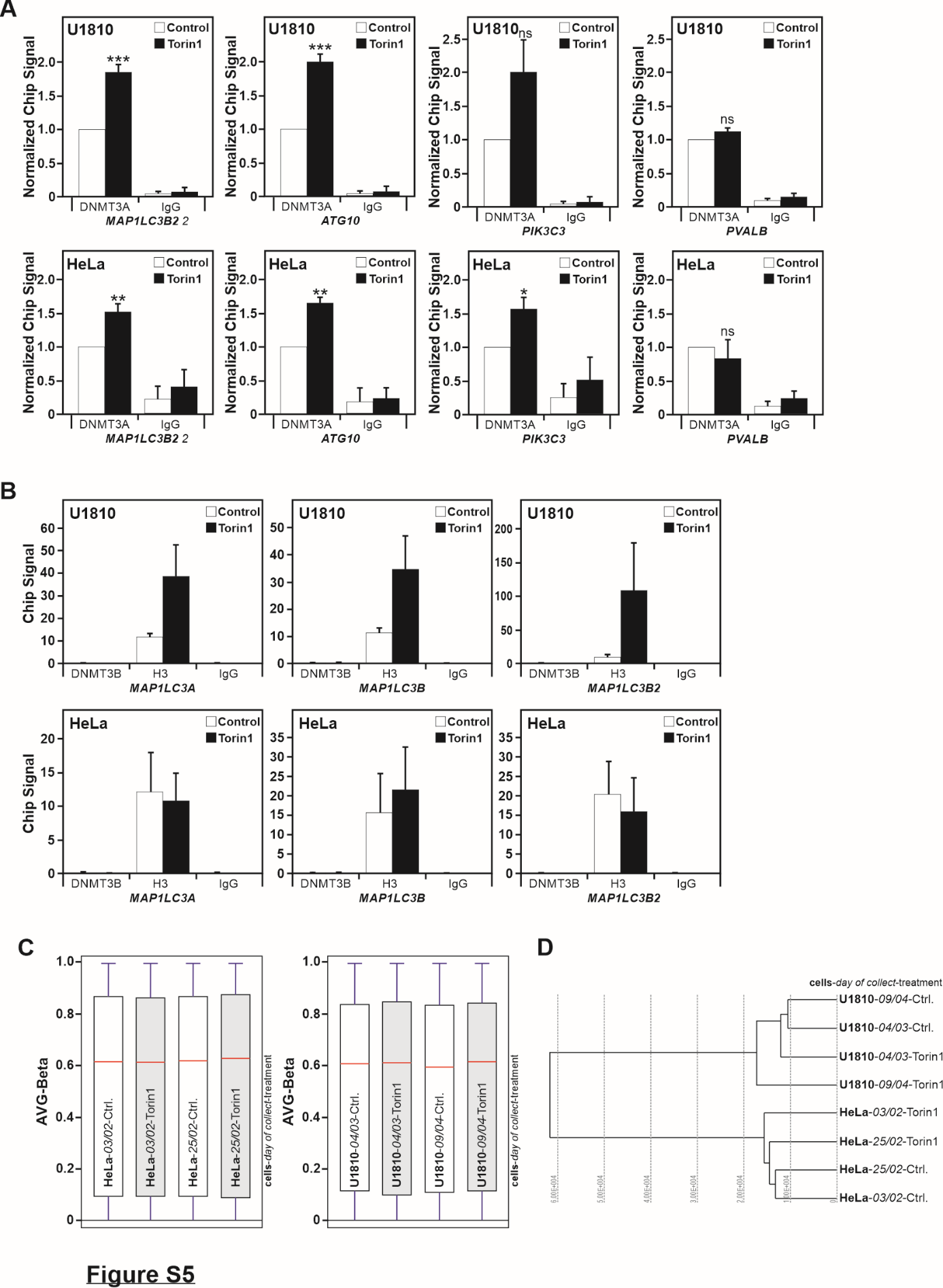


**Figure S5**. DNMT3A (but not DNMT3B) recruitment at the *MAP1LC3* loci in response to autophagy stimulation and genome-wide DNA methylation profiles of cells previously exposed to a transient autophagy stimulus. (**A**) Chromatin immunoprecipitation (ChIP) analysis of DNMT3A recruitment on the *MALP1LC3B2*, *ATG10*, *PIK3C3* and *PVALB* loci upon induction of autophagy with torin1 in HeLa cells for 6 h and U1810 cells for 2 h. (**B**) ChIP analysis of DNMT3B recruitment on the *MALP1LC3A, MALP1LC3B, MALP1LC3B2*, and *GAPDH* loci for the samples described in panel A. (**A** and **B**) All values are means of 3 independent experiments ± SEM and considered significant for *p<0.05, **p<0.01, ***p<0.001. n.s., not significant for the indicated comparison. (**C** and **D**) High-throughput profiling of the DNA methylation status of CpG (cytosine-guanine dinucleotide) islands was performed in HeLa and U1810 cells following a one-month recovery-period after an initial 250 nM torin1 or DMSO (used as control) treatment for 4 h. (**C**) Average methylation levels (Beta values) in the CpG islands for each sample are shown as box plots. (**D**) Cluster analysis of results illustrating a treatment rather than association with the collection dates is depicted.

**
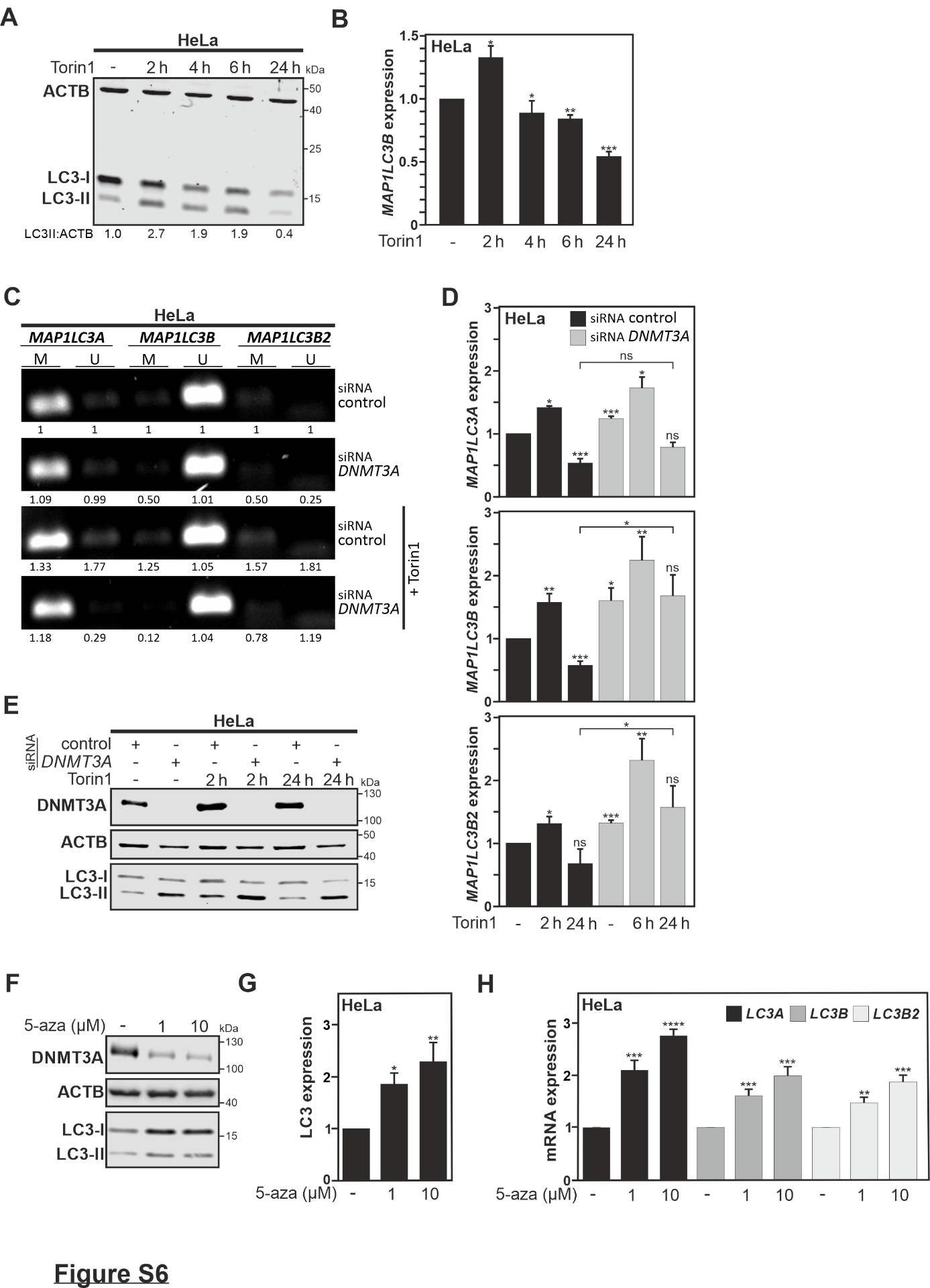
**

**Figure S6**. Impact of DNMT3A knockdown, or 5-Aza treatment on the expression of *MAP1LC3* isoforms. (**A**) Immunoblot analysis of LC3 expression in HeLa cells treated with torin1 for the indicated time points. (**B**) *MAP1LC3B* mRNA expression analyzed by RT-qPCR in HeLa cells treated as above. (**C**) Methylation-specific (MS)-PCR analysis of DNA methylation level at *MAP1LC3A*, *MAP1LC3B* and *MAP1LC3B2* loci in HeLa cells transfected with a siRNAs pool targeting *DNMT3A* expression, or a control siRNAs pool for 48 h and thereafter treated with torin1 or DMSO as control for 24 h. (**D**) Analysis of *MAP1LC3A*, *MAP1LC3B* and *MAP1LC3B2* mRNA expression levels by RT-qPCR in *DNMT3A*-siRNAs or *control*-siRNAs transfected HeLa cells treated with torin1 for 2 or 24 h. (**E**) Immunoblot analysis of LC3 in HeLa cells treated as in panel D. (**F**) Immunoblot analysis of DNMT3A and LC3 and (**G**) quantification of LC3 expression versus ACTB in HeLa cells treated with 1 µM or 10 µM 5-Aza, or DMSO used as control for 48 h. (**H**) Analysis of *MAP1LC3A*, *MAP1LC3B* and *MAP1LC3B2* mRNA expression levels by RT-qPCR in HeLa cells treated as in panel (F-G). All values are means of at least 3 independent experiments ± SEM and considered significant for *p<0.05, **p<0.01, ***p<0.001, ****p<0.0001. n.s., not significant for the indicated comparison. (n=3-4).


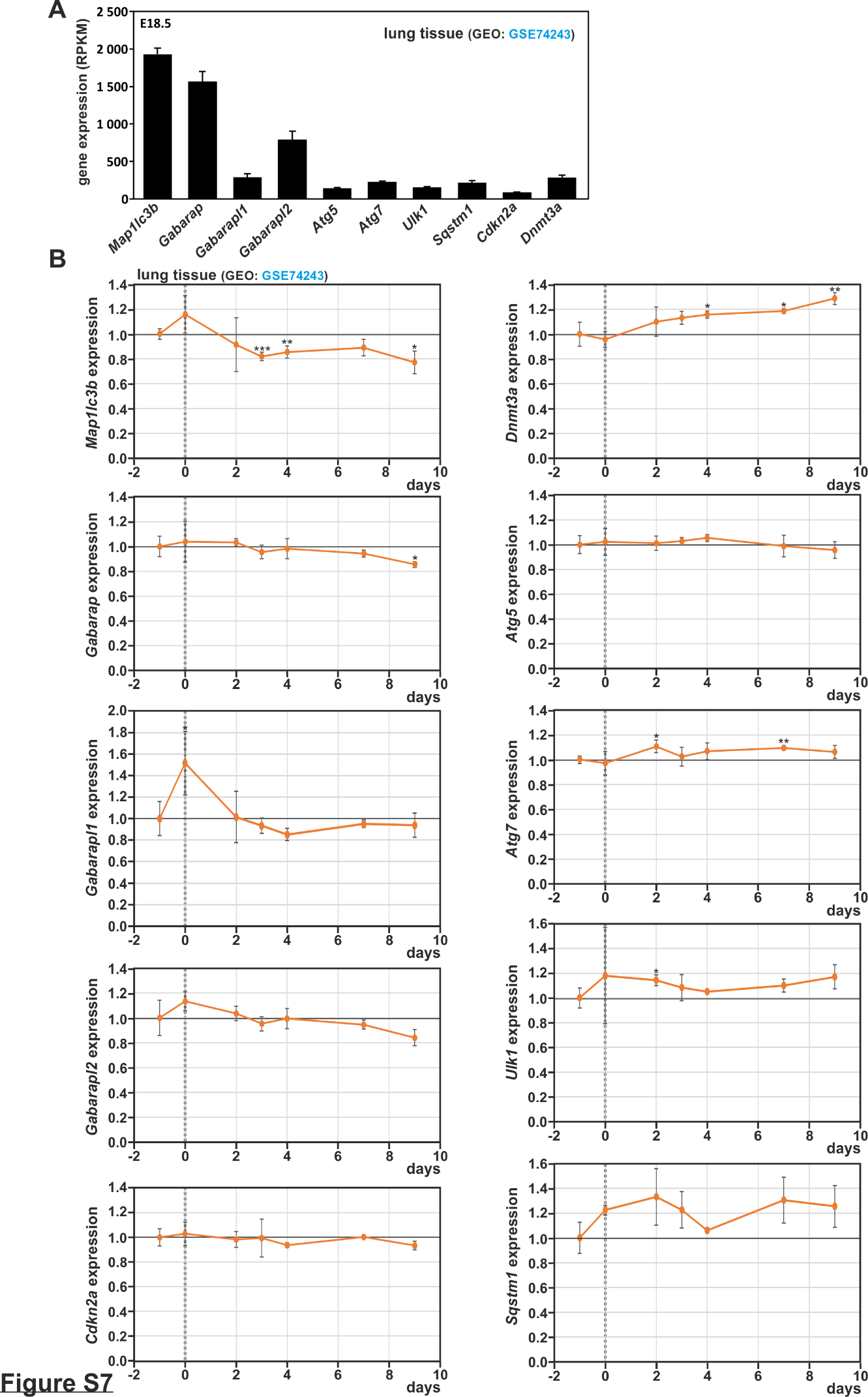


**Figure S7**. Analysis of the expression levels of autophagy-related genes in lung mouse tissues before and after placenta withdrawal. (**A**) Analysis of *Map1lc3b*, *Gabarapl1*, *Gabarapl2*, *Gabarap*, *Atg5*, *Atg7*, *Ulk1*, *Sqstm1*, *Cdkn2a*, *Dnmt3a* gene expression in mouse liver tissues at embryonic day (E)18.5 expressed as reads per kilobase of transcript per million mapped reads (RPKM), a normalized unit of transcript expression. (**B**) Analysis of the expression of the same genes from E18.5 to postnatal day 9, with 0 corresponding to the time of birth. Data are expressed as fold increase of E18.5 expression levels. All values are a mean of at 3 biological replicates ± SEM and considered significant for *p<0.05, **p<0.01, ***p<0.001, n.s., not significant for the indicated comparison.

**Table S1.** List of the siRNA sequences used in the study. Pool of siRNA sequences for *DNMT3A* and Non-targeting control used are provided.

| ON-TARGET plus SMARTpools siRNAs | Companies |
| --- | --- |
|  |  |
| *DNMT3A* (human, *DNMT3A* NM_022552) |  |
| GCAUUCAGGUGGACCGCUA | Dharmacon (L-006672) |
| GCACUGAAAUGGAAAGGGU |  |
| CUCAGGCGCCUCAGAGCUA |  |
| GGGACUUGGAGAAGCGGAG |  |
|  |  |
| *ATG5* (human, *ATG5* NM_004849) |  |
| GGCAUUAUCCAAUUGGUUU | Dharmacon (L-004374) |
| GCAGAACCAUACUAUUUGC |  |
| UGACAGAUUUGACCAGUUU |  |
| ACAAAGAUGUGCUUCGAGA |  |
|  |  |
| *ATG7* (human, *ATG7* NM_006395) |  |
| CCAACACACUCGAGUCUUU | Dharmacon (L-020112) |
| GAUCUAAAUCUCAAACUGA |  |
| GCCCACAGAUGGAGUAGCA |  |
| GCCAGAGGAUUCAACAUGA |  |
|  |  |
| Non-targeting siRNA pool |  |
| UGGUUUACAUGUCGACUAA | Dharmacon (D-001810) |
| UGGUUUACAUGUUGUGUGA |  |
| UGGUUUACAUGUUUUCUGA |  |
| UGGUUUACAUGUUUUCCUA |  |
| UGGUUUACAUGUUUUCCUA |  |

**Table S2.** List of the primers used for RT-qPCR. Sequences of PCR primers for quantitation of mRNA levels or ChIP. All the sequences are given 5’ to 3’.

| cDNA (organism) | Forward Primer | Reverse Primer |
| --- | --- | --- |
| Primers used for qPCR | | |
| *ACTB*  (human) | gatcaagatcattgctcctc | ttgtcaagaaagggtgtaac |
| *ATG10*  (human) | ggaagaagatgaggttcattgg | tcctttgatggtctccattc |
| *ATG13*  (human) | ctgaagacttggactcattac | ggactcaaggatacttttactg |
| *DNMT3A*  (human) | gaagagaagaatccctacaaag | caataatctccttgaccttgg |
| *GABARAP*  (human) | ttctgatctcacagttggtc | caagaaaaacaaggcatcc |
| *GABARAPL1*  (human) | gacgccttattcttctttgtc | catgattgtcctcatacagttg |
| *GAPDH*  (human) | acagttgccatgtagacc | tttttggttgagcacagg |
| *RB1CC1*  (human) | gcttgatccaaggattattcg | ctttctgttcattcaccagtc |
| *PI3KC3*  (human) | gtactggagaaaatgaaactgg | ccaatatacaaaggttgctcc |
| *ULK1*  (human) | attaacaagaagaacctcgc | cagtactccataaccaggtag |
| *GABARAPL2* primer set  (human) | Qiagen PPH07417A, sequence under disclosure | |
| *MAP1LC3A primer set*  (human) | Qiagen PPH19436A, sequence under disclosure | |
| *MAP1LC3B* primer set  (human) | Qiagen PPH17765B, sequence under disclosure | |
| *MAP1LC3B2* primer set  (human) | Qiagen PPH73070A, sequence under disclosure | |
| *SQSTM1* primer set  (human) | Qiagen PPH02107A, sequence under disclosure | |
| *B2M* primer set  (human) | Qiagen PPH01094E, sequence under disclosure | |
| *Dnmt3a*  (mouse) | ggcatggactgtggtcatgag | caatgatctccttgaccttag |
| *Gapdh*  (mouse) | tgcaccaccaattgcttagc | ggcatggactgtggtcatgag |
| *Map1lc3b*  (mouse) | gctcatcaagataatcagacg | gcataaaccatgtacaggaag |
| *dnmt3a*  (Zebrafish) | gctaagtttggtaaagtgcgg | ggatgtcctccttatcattca |
| *map1lc3a*  (Zebrafish) | gtaaagattatcaggcgtcg | gctgattgacaagaaggaaa |
| *map1lc3b*  (Zebrafish) | gaacagcatccaaacaagat | ttgcttctctcccttgtatc |
| *sqstm1*  (Zebrafish) | agtgagggaacaaagaaaga | ctaagatgagtccattcctca |
| *actb*  (Zebrafish) | gtatgcagaaggaaatcacc | tcttgatcttcatggtggaa |
|  | | |
| Primers used for ChIP | | |
| CpG *ATG10*  (human) | tcgctgcacagtactcagtg | aacagatcctgagttgcccc |
| CpG *MAP1LC3A*  (human) | tggctggaaaggtcaaggtc | gagtatgcagctgtgagggg |
| CpG *MAP1LC3B*  (human) | gtcgccacagacgacctaac | gtttgcgcaactgcttttgt |
| CpG *MAP1LC3B2.1*  (human) | tgtgtcttaatagaaggcacccc | ttttggggtcttattcaagccttt |
| ChIP *MAP1LC3B2*  (human) | ccttgtaaagggcgatagca | tagctccatcctacgcatcc |
| CpG *PI3KC3*  (human) | attagacagctagcggggca | atctctaaaccctctgagctcct |
| ChIP *PVALB*  (human) | Human ChIP-seq grade Myoglobin Exon 2 primer pair  (Diagenode; C170110006); Sequence under disclosure | |
| Primers used for MS-PCR | | |
| Methylated *MAP1LC3A*  (human) | ggatatttacgagtaggagaaagac | cccctactactaactcaaaaaccg |
| Unmethylated *MAP1LC3A*  (human) | ggatatttatgagtaggagaaagat | ccctactactaactcaaaaaccaaaa |
| Methylated *MAP1LC3B*  (human) | gtttagaatgaaggttcggga | ataaccgctaataacctccgc |
| Unmethylated *MAP1LC3B*  (human) | gtttagaatgaaggtttggga | aaataaccactaataacctccaccc |
| Methylated *MAP1LC3B2*  (human) | gtcggagaagatttttaagtagcg | ttaaatactactctcgaataaatcgaac |
| Unmethylated *MAP1LC3B2*  (human) | tggagaagatttttaagtagtgg | aattaaatactactctcaaataaatcaaac |
